# Synthesis, Curing and Thermal Behavior of Amine Hardeners from Potentially Renewable Sources

**DOI:** 10.3390/polym15040990

**Published:** 2023-02-16

**Authors:** Torben Wiegand, Andrea Osburg

**Affiliations:** F. A. Finger-Institute for Building Material Engineering, Chair of Construction Chemistry and Polymer Materials, Bauhaus-Universität Weimar, Coudraystraße 11A, 99423 Weimar, Germany

**Keywords:** epoxy, amine hardener, curing agent, vanillin, syringaldehyde, aniline, renewable, DSC, TG-MS, bio-based

## Abstract

Research into bio-based epoxy resins has intensified in recent decades. Here, it is of great importance to use raw materials whose use does not compete with food production. In addition, the performance of the newly developed materials should be comparable to that of conventional products. Possible starting materials are lignin degradation products, such as vanillin and syringaldehyde, for which new synthesis routes to the desired products must be found and their properties determined. In this article, the first synthesis of two amine hardeners, starting with vanillin and syringaldehyde, using the Smiles rearrangement reaction is reported. The amine hardeners were mixed with bisphenol A diglycidyl ether, and the curing was compared to isophorone diamine, 4-4′-diaminodiphenyl sulfone, and 4-Aminonbenzylamine by means of differential scanning calorimetry. It was found that the two amines prepared are cold-curing. As TG-MS studies showed, the thermal stability of at least one of the polymers prepared with the potentially bio-based amines is comparable to that of the polymer prepared with isophorone diamine, and similar degradation products are formed during pyrolysis.

## 1. Introduction

The use of epoxy resins in a wide variety of applications, such as coatings, polymer concrete and infrastructure constructions, is becoming increasingly important [1,2,3]. In addition, fundamental studies on curing behavior and network formation of epoxy resins are still the subjects of current research [4]. Efforts are being made to become independent of petroleum-based materials and to use starting materials from renewable sources [5]. Since lignin, in particular, is produced in large quantities as a by-product in paper production and is currently mainly used as a heating fuel, it is a viable raw base material [6]. The extraction of phenols such as vanillin and syringaldehyde from various wood species is well established [7], and the extraction of basic chemicals from lignin is still the subject of research [8,9]. The use of large quantities of lignin does not compete with food production, as may be the case with other renewable raw materials such as vegetable oils [10]. One possibility is using the aromatics obtained in the production of bio-based epoxy resins. A lot of research has been conducted in this field over the past decades [11,12,13,14].

The synthesis of an aliphatic amine starting from vanillin was already described one century ago [15], but the compound has not been studied as an amine hardener for epoxy resins until 2015 [16], and since then, different epoxy resin components have been prepared from vanillin and their properties have been explored [17,18]. However, the presence of an additional amine moiety for a higher degree of cross-linking in the epoxy resin and, consequently, higher mechanical and thermal stability would be an advantage. The aromatic hydroxyl group also reacts with still present oxiranes at elevated temperatures [19], but since only one epoxy moiety can be added, the degree of cross-linking is not increased, especially with incomplete curing. Due to the fact that a second epoxy group can be linked to amines, the conversion of phenols into anilines is consequential.

The Smiles rearrangement is an intramolecular, nucleophilic aromatic substitution reaction, which was described in 1931 [20]. It was later used to transform phenols into anilines [21,22,23]. For the Smiles rearrangement, the presence of an acid amide that acts as a transfer reagent is the requirement, and electron-withdrawing substituents on the phenol promote the reaction. In order to obtain an additional reactive unit for the cross-linking of epoxy resins, it is possible to convert the aldehyde moiety into the corresponding oxime [24] and, subsequently, use different reducing agents for the respective amine [25,26].

**Figure 1:**
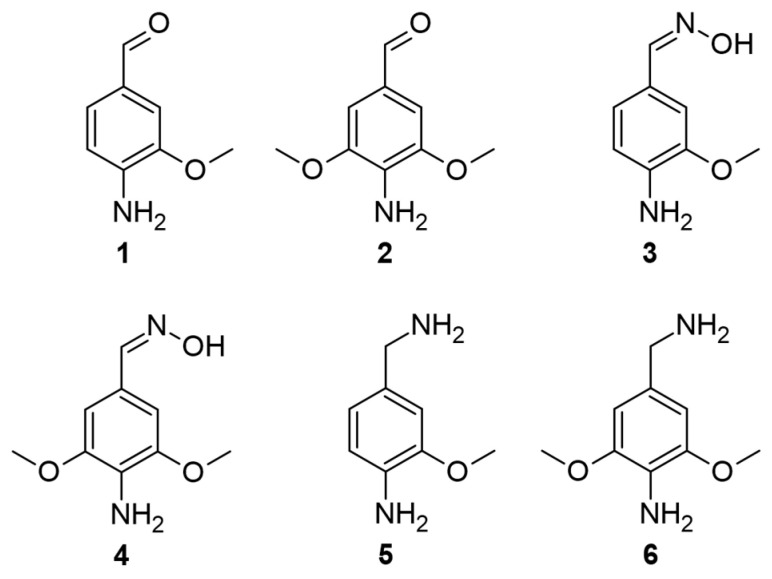
Amines **1**–**6** are synthesized from renewable resources.

**Figure 2:**
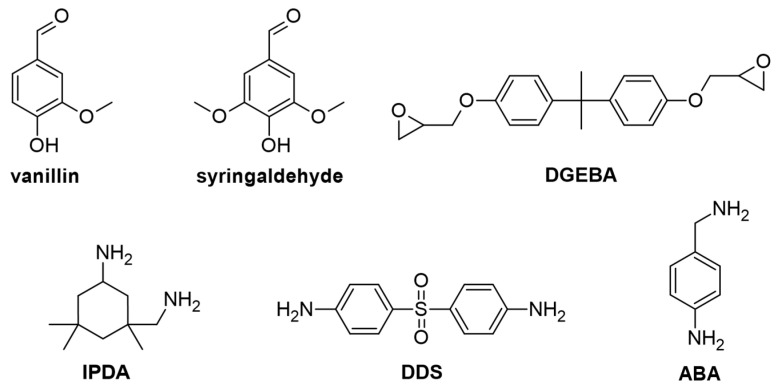
Starting materials (vanillin and syringaldehyde) for the synthesized amine hardeners and the reference epoxy resin components DGEBA, IPDA, and DDS, as well as ABA used for comparative purposes.

In this paper, the first direct synthesis of six aromatic amines **1**–**6** (Figure 1) from vanillin and syringaldehyde (Figure 2) available from renewable resources is reported.

Since compounds **5** and **6** contain two amine moieties, it is possible to use them as curing agents for epoxy resins. For the curing behavior studies, diglycidyl ether of bisphenol A (DGEBA, Figure 2) was used as the epoxy component. Since aromatic and aliphatic amine moieties are present in compounds **5** and **6**, both the cold-curing isophorone diamine (IPDA, Figure 2) and the hot-curing 4-4′-diaminodiphenyl sulfone (DDS, Figure 2) were used for comparison. The reactivity of compounds also depends on the type, position, and the number of substituents [27]. Since vanillin and syringaldehyde and the amines formed from them differ by one methoxy moiety, the curing behavior of the unsubstituted 4-aminobenzylamine (ABA, Figure 2) was also investigated for comparative purposes.

The curing behavior of possible new amine hardeners for epoxy resins can be determined by means of differential scanning calorimetry (DSC). Conclusions can be drawn about the curing behavior, especially in comparison to known amine hardeners [28].

Thermogravimetric analysis coupled with mass spectrometry (TG-MS) is suitable for investigating the behavior of polymer materials at elevated temperatures, and gaseous degradation products can be identified [29].

## 2. Materials and Methods

### 2.1. General

**Scheme 1:**
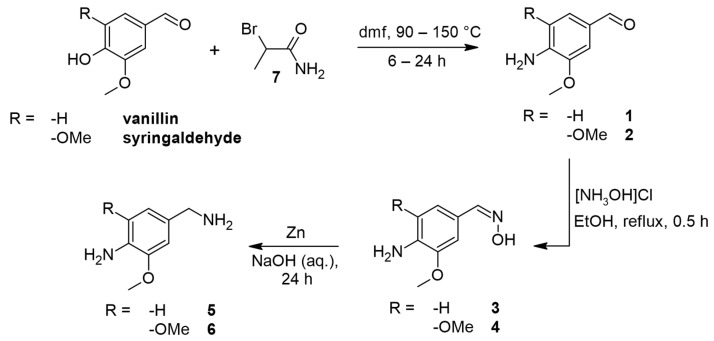
Synthetic route to compounds **1**–**6**.

Vanillin, thionyl chloride, zinc dust, and all solvents were purchased from Carl Roth GmbH + Co. KG, Karlsruhe, Germany. Syringaldehyde, sodium hydroxide, potassium carbonate, potassium iodide, hydroxylamine hydrochloride, sodium acetate trihydrate, bisphenol A diglycidyl ether (DGEBA), isophorone diamine (IPDA) and 4-aminobennzylamine (ABA) were purchased from VWR international GmbH, Darmstadt, Germany. All materials were used without further purification. Silica gel 60 (0.035–0.07 mm, 400–220 mesh) and pre-coated thin-layer chromatography (TLC) plates (layer: 0.20 mm, silica gel 60) were used for column chromatography. The compound, 2-bromopropionic acid amide (**7**, Scheme 1), was prepared from the corresponding acid using a procedure described in the literature [30].

^1^H and ^13^C (proton decoupled) NMR spectra were recorded on Bruker (Billerica, MA, USA) Avance 200, 400, and 600 spectrometers. The chemical shifts are reported in ppm and refer to tetramethylsilane. Infrared (IR) spectra were obtained with a Thermo Fisher Scientific (Waltham, MA, USA) iZ10. Elemental analyses were performed with a Vario EL III CHNS (Elementar Analysesysteme GmbH, Langenselbold, Germany). Mass spectra were obtained with an Agilent (Santa Clara, CA, USA) 5977B MSD and a Finnigan MAT (Bremen, Germany) SSQ 7000. All DSC studies of the curing behavior and the determination of melting points (extrapolated onset temperatures) were performed with the TA Instruments (New Casle, DE, USA) DSC Q200 differential scanning calorimeter under a nitrogen atmosphere. High-purity sapphire and indium were used for calibration. Thermogravimetric analysis coupled with mass spectrometry (TG-MS) was carried out using an STA 449 F3 Jupiter instrument (Netzsch, Selb, Germany) coupled online with a quadrupole mass spectrometer QMS 403 D Aëolus (Netzsch, Selb, Germany).

### 2.2. Synthesis of 4-Amino-3-methoxy benzaldehyde (***1***)

To a heated suspension (90 °C) of vanillin (10.2 g; 1 eq.), K_2_CO_3_ (24 g; 2 eq.), and KI (2.5 g; 0.25 eq.) in 100 mL of *N,N*-dimethylformamide (dmf), a suspension of 2-bromo propionic acid amide (11.5 g; 1.1 eq.) in 100 mL of dmf was added. The mixture was stirred at 90 °C for two hours, and solid NaOH (24.6 g; 9 eq.) was added. The resulting mixture was heated to reflux (153 °C) for four hours. Strong gas evolution was observed at about 120 °C. After cooling, sufficient amounts of water (200 mL) and dichloromethane (200 mL) were added to obtain two separate layers. After four extractions with dichloromethane, the combined organic layers were washed four times with water, dried over anhydrous magnesium sulfate, and the solvent was removed under reduced pressure. After column chromatography using dichloromethane as the solvent, 1.28 g (13%) of a bright yellow solid was obtained.

M.p. 99 °C. ^1^H NMR (CDCl_3_), δ, ppm: 9.73 (s, 1H), 7.33 (m, 1H), 7.30 (s, 1H), 6.73 (d, 2H), 4.52 (s, 2H), 3.91 (s, 3H); ^13^C NMR (CDCl_3_): *δ* 190.7, 146.8, 143.3, 128.1, 127.6, 112.7, 108.3, 55.7. MS (DEI): *m*/*z* = 151 (M^+^, 100%), 136 (M^+^-CH3, 70%). IR (KBr): *ν* (cm^−1^) = 3446 (s), 3344 (s), 3221 (m), 3002 (w), 2967 (w), 2941 (w), 2855 (w), 2838 (w), 1648 (s), 1621 (s), 1587 (s), 1571 (s), 1519 (s), 1466 (m), 1456 (m), 1436 (m), 1323 (s), 1249 (s), 1200 (m), 1157 (m), 1138 (s), 1029 (s), 855 (m), 803 (s), 726 (m), 629 (m), 584 (m), 545 (m). Anal. Calc. for C_8_H_9_NO_2_: C, 63.56; H, 6.00; N, 9.27. Found: C, 63.52; H, 5.95; N, 9.47.

### 2.3. Synthesis of 4-Amino-3,5-dimethoxy benzaldehyde (***2***)

A suspension of syringaldehyde (5.1 g; 1 eq.), K_2_CO_3_ (10.0 g; 2.6 eq.), and KI (2.33 g; 0.5 eq.) in 90 mL of dmf was heated to 90 °C. After the addition of 2-bromopropionic acid amide (4.8 g; 1.1 eq.), dissolved in 50 mL of dmf, the heated mixture was stirred for 16 h. To the resulting dark red suspension, NaOH (10.25 g; 9 eq.) was added, and the mixture was refluxed (153 °C) for 8 h. After cooling, sufficient amounts of water 100 mL) and dichloromethane (100 mL) were added to obtain two separate layers. After four extractions with dichloromethane, the combined organic layers were washed four times with water and dried over anhydrous magnesium sulfate. The solvent was removed under reduced pressure, and a red oil was obtained. After column chromatography using dichloromethane as the solvent, 0.3 g (6%) of a bright yellow solid was obtained.

M.p. 94 °C. ^1^H NMR (CDCl_3_), δ, ppm: 9.71 (s, 1H), 7.06 (s, 2H), 4.48 (s, 2H), 3.91 (s, 6H); ^13^C NMR (CDCl_3_), δ, ppm: 190.7, 146.3, 133.0, 125.6, 106.2, 56.0. MS (DEI): *m/z* = 181 (M^+^, 100%), 166 (M^+^-CH_3_, 90%). IR (KBr): *ν* (cm^−1^) = 3462 (s), 3350 (s), 3176 (m), 3013 (w), 2993 (m), 2965 (m), 2938 (m), 2836 (m), 2793 (w), 1667 (s), 1605 (s), 1591 (s), 1570 (s), 1515 (s), 1470 (s), 1451 (m), 1432 (m), 1408 (w), 1368 (m), 1335 (s), 1279 (m), 1195 (m), 1161 (s), 1059 (m), 1039 (m), 986 (m), 864 (m), 841 (m), 722 (m), 614 (m), 581 (m), 522 (w), 444 (m). Anal. Calc. for C_9_H_11_NO_3_: C, 59.66; H, 6.12; N, 7.73. Found: C, 59.61; H, 6.17; N, 7.45.

### 2.4. Synthesis of 4-Amino-3-methoxy benzaldehyde oxime (***3***)

A solution of benzaldehyde **1** (1.06 g; 1 eq.), hydroxylamine hydrochloride (0.73 g, 1.5 eq.), and sodium acetate trihydrate (2.4 g, 2.5 eq.) in 18 mL of water and 6 mL of ethanol was heated to reflux for 0.5 h. The formed solution was poured on ice, and the colorless precipitate was filtered off, thoroughly washed with cold water, and recrystallized from boiling ethanol. After drying in a vacuum, 1.02 g (89%) of a colorless solid was obtained.

M.p. 134 °C. ^1^H NMR (thf-d8), δ, ppm: 9.62 (s, 1H), 7.86 (s, 1H), 7.09 (s, 1H), 6.82 (d, 1H), 6.54 (d, 1H), 4.57 (s, 2H), 3.79 (s, 3H); ^13^C NMR (thf-d8), δ, ppm: 149.4, 147.8, 140.2, 123.2, 122.0, 113.8, 108.1, 55.6. MS (DEI): *m*/*z* = 166 (M^+^, 100%), 151 (M^+^-CH3, 80%). IR (KBr): *ν* (cm^−1^) = 3386 (s), 3287 (s), 3159 (m), 3131 (m), 3050 (m), 3017 (m), 2983 (m), 2969 (m), 2936 (m), 2869 (s), 2830 (s), 2772 (s), 2628 (w), 1616 (m), 1588 (m), 1518 (s), 1470 (m), 1449 (m), 1422 (m), 1359 (m), 1316 (m), 1283 (s), 1267 (m), 1241 (m), 1194 (m), 1161 (m), 1102 (m), 1035 (m), 972 (w), 945 (s), 896 (m), 860 (s), 823 (m), 787 (m), 756 (s), 726 (w), 623 (m), 609 (m), 552 (w), 530 (w), 473 (w), 460 (w), 440 (m), 412 (w). Anal. Calc. for C_8_H_10_N_2_O_2_: C, 57.82; H, 6.07; N, 16.82. Found: C, 57.91; H, 6.15; N, 16.92.

### 2.5. Synthesis of 4-Amino-3,5-dimethoxy benzaldehyde oxime (***4***)

To a solution of benzaldehyde **2** (0.22 g; 1 eq.) in 3 mL of water and 1 mL of ethanol, hydroxylamine hydrochloride (0.13 g; 1.5 eq.) and sodium acetate trihydrate (0.42 g, 2.5 eq.) were added. The resulting suspension was heated to reflux for 0.5 h. The solution was poured on ice. The formed white precipitate was filtered and washed with cold water. After recrystallization from boiling ethanol and drying in a vacuum, 0.19 g (81%) of a colorless solid was obtained.

M.p. 147 °C. ^1^H NMR (thf-d8), δ, ppm: 9.64 (s, 1H), 7.86 (s, 1H), 6.77 (s, 2H), 4.26 (d, 2H), 3.80 (d, 6H); ^13^C NMR (thf-d8), δ, ppm: 149.5, 147.5, 129.0, 122.0, 103.7, 55.9. MS (DEI): *m*/*z* = 196 (M^+^, 100%), 181 (M^+^-CH3, 50%). IR (KBr): *ν* (cm^−1^) = 3380 (s), 3282 (s), 3153 (m), 3128 (m), 3084 (m), 3049 (m), 2969 (m), 2939 (m), 2913 (m), 2870 (m), 2846 (m), 2799 (m), 2755 (m), 2736 (m), 1601 (s), 1514 (s), 1453 (m), 1419 (m), 1362 (w), 1335 (s), 1297 (w), 1244 (m), 1197 (s), 1176 (s), 1156 (m), 1097 (s), 1046 (w), 959 (m), 937 (w), 912 (m), 873 (m), 837 (m), 821 (m), 794 (m), 723 (m), 642 (w), 618 (m), 527 (w), 444 (w). Anal. Calc. for C_9_H_12_N_2_O_3_: C, 55.09; H, 6.16; N, 14.28. Found: C, 54.91; H, 6.18; N, 14.22.

### 2.6. Synthesis of 4-Amino-3-methoxy benzylamine (***5***)

In a 25-mL flask, 270 mg (1.62 mmol) of oxime **3**, 0.3 g of NaOH, and 1.3 g of zinc dust were placed. Then, 7 mL of water was added, the flask was sealed with a bubble counter, and the suspension was stirred at room temperature for 24 h. The mixture was then filtered, and the filter cake was extracted with dichloromethane. The filtered aqueous layer was also extracted three times with dichloromethane. The combined organic layers were washed three times with water and dried over anhydrous sodium sulfate. After the solvent was removed under reduced pressure and the product was dried overnight in a vacuum, 110 mg (44%) of a colorless solid was obtained.

M.p. 73 °C. ^1^H NMR (thf-d8), δ, ppm: 6.85 (d, 1H), 6.67 (dd, 1H), 6.58 (dd, 1H), 4.20 (bs, 2H), 3.84 (s, 3H), 3.71 (s, 2H), 1.56 (bs, 2H); ^13^C NMR (thf-d8), δ, ppm: 146.9, 136.0, 133.1, 119.2, 113.4, 109.3, 54.6, 46.5. MS (DEI): *m*/*z* = 152 (M^+^, 100%), 136 (M^+^-NH_2_, 90%). IR (KBr): *ν* (cm^−1^) = 3443 (m), 3345 (m), 3186 (m), 3004 (w), 2938 (w), 2916 (w), 2859 (w), 2835 (w), 1620 (m), 1586 (m), 1517 (s), 1463 (m), 1450 (m), 1424 (m), 1347 (m), 1281 (s), 1238 (s), 1193 (m), 1144 (s), 1072 (m), 1031 (s), 956 (m), 904 (s), 863 (s), 811 (m), 794 (m), 735 (m), 709 (m), 635 (s), 553 (m).

### 2.7. Synthesis of 4-Amino-3,5-dimethoxy benzylamine (***6***)

In accordance with the synthesis of benzylamine **5**, a suspension of 260 mg (1.32 mmol) of oxime **4**, 0.3 g of NaOH, and 1.1 g of zinc dust in 7 mL of water were stirred at room temperature for 24 h. After filtering and washing the residue with dichloromethane, the aqueous layer was extracted three times with dichloromethane. The combined organic layers were washed three times with water, dried over anhydrous sodium sulfate, and the solvent was removed under reduced pressure. After drying in a vacuum overnight, 85 mg (35%) of colorless oil was obtained.

^1^H NMR (thf-d8), δ, ppm: 6.58 (s, 2H), 3.92 (bs, 2H), 3.84 (s, 6H), 3.73 (s, 2H), 1.99 (bs, 2H); ^13^C NMR (CD_2_Cl_2_), δ, ppm: 148.0, 133.3, 124.7, 103.8, 56.5, 47.4. MS (DEI): *m*/*z* = 182 (M^+^, 100%), 166 (M^+^-NH_2_, 85%). IR (KBr): *ν* (cm^−1^) = 3450 (w), 3361 (w), 2996 (w), 2935 (w), 2836 (w), 1599 (m), 1513 (m), 1456 (m), 1426 (m), 1382 (w), 1320 (m), 1254 (w), 1186 (m), 1154 (s), 1070 (m), 942 (w), 824 (m), 744 (w), 661 (m), 618 (m), 571 (m).

### 2.8. Thermal Analyses

The synthesized amine hardeners **5** and **6,** as well as 4-aminobenzylmine, were mixed with DGEBA in order to investigate the curing and thermal stability of the resulting epoxy resins. Since both aliphatic and aromatic moieties are contained in the synthesized amines, both IPDA, which cures with DGEBA at room temperature, and DDS, a curing agent for hot-curing systems, are used as reference curing agents.

The reaction of primary amines with epoxy moieties has already been studied in detail. It is well-known that two epoxy moieties can be linked to a single primary amine moiety [28,31,32]. Therefore, DGEBA was mixed with the amine hardeners in such a way that two epoxy groups were added to one amine moiety. To ensure complete melting of the DGEBA before mixing, it was stored for 12 h at 45 °C. After weighing, the finely ground solid amines, as well as oily hardener **6** and the liquid DGEBA, were intensively stirred manually.

For DSC analyses, all samples (20–30 mg) were contained within covered but not tightly sealed aluminum DSC pans. The heating rate for all scanning runs was 10 K/min. The maximum temperatures of the DSC measurements were 250 °C in nearly all cases. Only when DDS was used as a curing agent, and to ensure complete curing, was the measurement carried out up to 320 °C. Glass transition temperatures (T_g_) of the formed polymers were determined as the inflection point of the heat capacity jump. For TG-MS analyses, about 30 mg of the materials were heated from 35 to 1000 °C with a heating rate of 10 K/min under an argon atmosphere (50 mL/min purge gas, 20 mL/min protective gas) using platinum crucibles. MS spectra of the gaseous degradation products ranging from 10 to 290 m/z were collected at the studied temperature interval.

## 3. Results and Discussion

### 3.1. Synthesis of the Amines

For the synthesis of aniline derivatives from the corresponding phenols vanillin and syringaldehyde, a combination of different commonly used methods proved appropriate [21,22,23]. This resulted in a two-step, one-pot synthesis in which 2-bromopropionic acid amide (**7**) was used as an amination agent for the Smiles rearrangement reaction, in which anilines **1** and **2** were formed (Scheme 1).

The Smiles rearrangement reaction is typically used for the conversion of phenols with electron-withdrawing substituents. However, vanillin and syringaldehyde contain one or even two electron-donating methoxy moieties. This results in low yields of thirteen percent for vanillin and six percent for syringaldehyde. GC-MS analyses of the reaction mixture still showed high amounts of starting materials, but an increase in the reaction time to a total of 24 h could not significantly increase the yields. However, the methoxy moiety also appears to have a stabilizing effect on the anilines formed. When 4-aminobenzaldehyde (no methoxy substituents) was reacted under the same conditions, a reddish insoluble solid was obtained. This could have formed due to a Schiff Base reaction of the targeted product [33]. The isolated aldehydes **1** and **2** could be transferred into the oximes **3** and **4** according to established methods using hydroxylamine hydrochloride (see Scheme 1) [34].

Finally, it was possible to obtain the amine hardeners **5** and **6** from the reaction of oximes **3** and **4** with zinc powder in an aqueous sodium hydroxide solution [26]. The purification was comparatively simple and afforded the desired amines in moderate yields.

### 3.2. DSC Analyses

**Figure 3:**
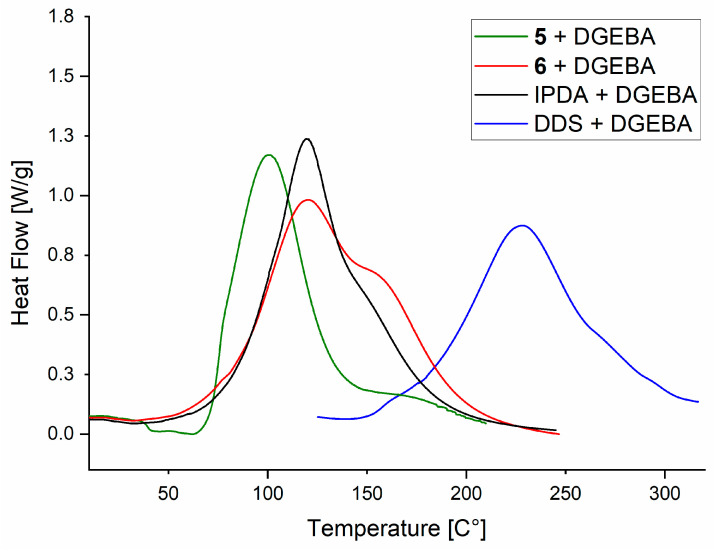
DSC thermograms of hardeners **1**, **2**, IPDA, and DDS during curing with DGEBA.

Figure 3 shows the DSC thermograms of amine hardeners **1** and **2** with DGEBA during curing compared to the combinations of IPDA and DDS with DGEBA.

It can be seen that the curing of the vanillin and syringaldehyde-based compounds starts in the same temperature range as the curing of the aliphatic reference hardener IPDA and is completed within the temperature range of a cold-curing amine. The onset temperatures of the curing of the investigated compounds are listed in Table 1. This suggests that initially, the aliphatic amine moiety reacts with the epoxy groups of DGEBA. The increase in the measured heat flow during curing of **6** and IPDA is initially comparatively low. In the case of compound **5**, no increase was measured initially, but the heat flow increased sharply from about 70 °C onwards. This is probably related to the difference in the state of matter of the substances studied. Compound **6** and IPDA are liquids, while **5** is a solid that melts at 73 °C.

Furthermore, it can be seen that the heat flow curves of the curing of **5** and **6** with DGEBA exhibit a tailing shoulder, indicating curing of the aromatic amine moiety taking place at higher temperatures. A comparison with the curing curve of DDS, which is a hot-curing amine, with DGEBA shows that the curing of the amine group of compounds **5** and **6** starts at lower temperatures than the curing of DGEBA with DDS.

**Figure 4:**
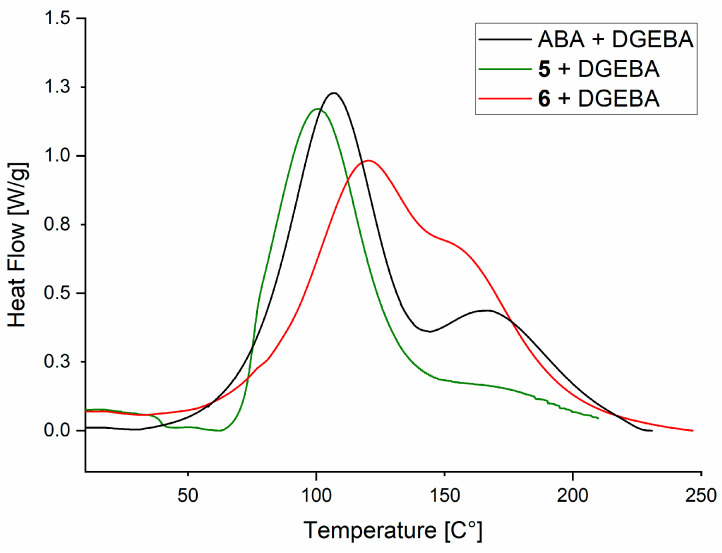
DSC thermograms of hardeners **1**, **2**, and ABA during curing with DGEBA.

Figure 4 compares the curing curves of compounds **5** and **6** with that of 4-aminobenzylamine (ABA). In all three curves, the superposition of the reaction heat generated during the curing of the aliphatic and aromatic amine moieties can be seen. However, in ABA, the distinction is most prominent. Furthermore, it can be seen that the different substitution pattern of the three compounds does not affect the reactivity significantly. Here, the state of the matter probably has a major impact on reactivity since the melting point of compound **5** is approximately in the range where the curing reaction starts, making it impossible to separate these two effects.

Table 1 also lists the glass transition temperatures of the polymers prepared. It can be clearly seen that the glass transition temperature of the polymer prepared from solid curing agents **5** and DGEBA shows the lowest value. Here, melting the components before mixing with DGEBA would certainly produce a more homogeneous resin-hardener mixture and thus increase T_g_. This suggests the behavior of hardener **6**, which is already liquid before the start of the curing reaction. The polymer formed with this shows a significantly higher glass transition temperature. However, since these are cold-curing amines, pre-heating would complicate the interpretation of the results. Furthermore, it can be seen that hardener **6** gives a higher glass transition temperature than IPDA and is thus well suited as a cold-curing amine hardener for comparatively temperature-stable epoxy resin formulations based on renewable resources. The cured polymers were amber to reddish in color.

### 3.3. TG-MS Investigations

**Figure 5:**
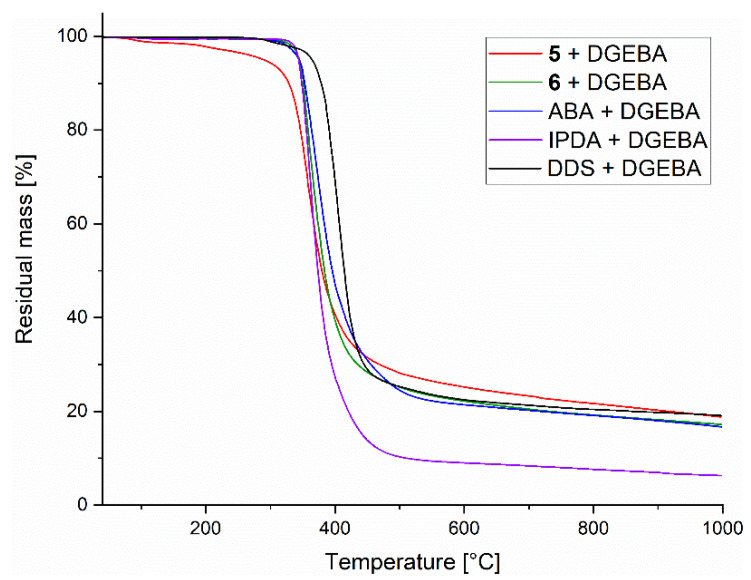
TGA curves of the investigated amines combined with DGEBA.

To investigate the thermal stability of the resins formed, the freshly produced amine-DGEBA combinations were also analyzed thermogravimetrically. The evolved gases during thermogravimetric analysis were investigated by mass spectroscopy. Due to the small sample quantity, at a heating rate of 10 K/min, complete curing of the epoxy resin was assumed before the thermal decomposition started. The thermogravimetric curve of DGEBA combined with IPDA shown in Figure 5 can be compared with the literature values in which the sample was previously heated [16]. Consequently, the gradual increase in temperature of 10 K/min results in sufficient tempering for almost identical thermal stability. Figure 5 also shows the TGA curve of the investigated amines combined with DGEBA.

**Figure 6:**
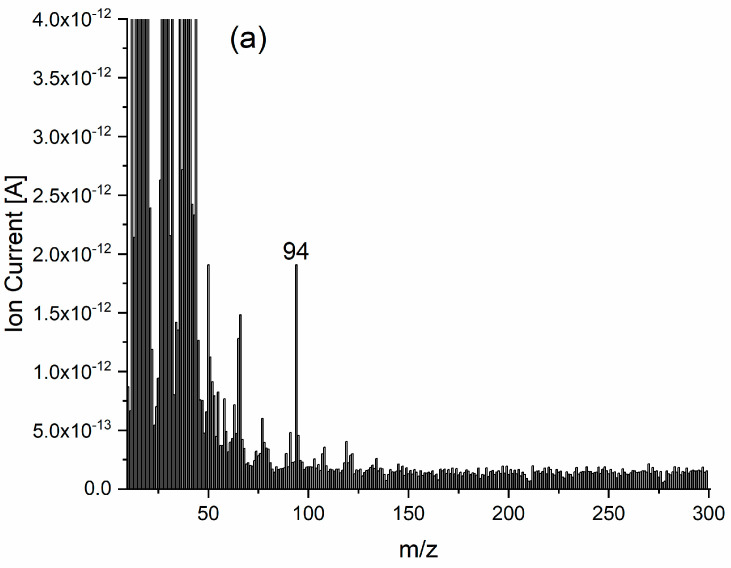

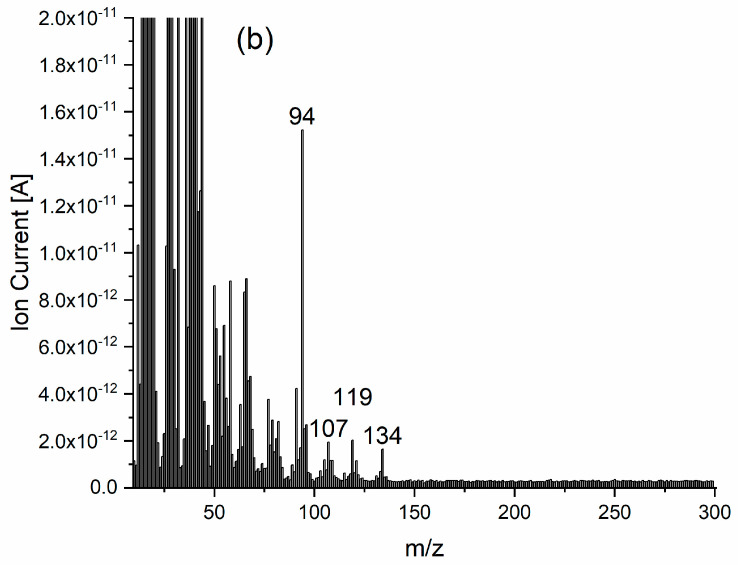
(**a**) MS spectrum of the gases evolved during TG measurement of the polymer formed from amine **5** and DGEBA at 379 °C and (**b**) MS spectrum of the gases evolved during TG measurement of the polymer formed from IPDA and DGEBA at 369 °C.

The inflection point of mass loss, or the maximum of the first derivative of mass loss, for the polymers prepared from the novel amine hardeners **5** and **6** shows similar values to that of the polymers prepared from ABA od IPDA. Only the polymer prepared from DDS and DGEBA has significantly higher thermal stability because it is a purely aromatic amine curing agent. However, when using diamine **5**, it can be seen that the loss of mass already starts at much lower temperatures than with all other amine hardeners used. This could be due to the formation of defects as a result of the poorer mixing of the solid with the liquid DGEBA. When IPDA is used as a curing agent, a significantly higher residual mass loss can be determined. As a consequence of the aliphatic structure of the amine hardener IPDA, gaseous pyrolysis products are formed more easily than from aromatic hydrocarbons. These gaseous pyrolysis products were analyzed using a coupled mass spectrometer. For example, Figure 6 shows the mass spectra obtained during the pyrolysis of the polymer prepared from amine **5** and DGEBA and the polymer prepared from IPDA and DGEBA. Since the numerous gaseous pyrolysis products are not separated before the mass spectroscopic measurement, the mass spectra of the simultaneously emitted compounds are superimposed.

In both mass spectra, a peak at *m*/*z* = 94 is detectable. Furthermore, peaks at *m*/*z* = 107, 119, and 134 are reliably detectable in Figure 6b. The detection of these peaks in Figure 6a is not reliable as the signal-to-noise ratio here is less than 3:1. The said peaks can be assigned to phenol (*m*/*z* = 94), as well as differently substituted derivatives of phenol [35]. Since these pyrolysis products are also formed during the decomposition of the IPDA-based polymer, it can be assumed that they originate at least in part from the DGEBA component.

## 4. Conclusions

Two differently substituted diamines were prepared to act as amine-curing agents for epoxy resins. For this purpose, a synthetic route was chosen that starts from vanillin and syringaldehyde. Since these two substances can be obtained from lignin, a by-product of paper production, it would be possible to produce the amine hardeners from renewable raw materials without competing against food production. For preparation, the Smiles rearrangement reaction was adopted, which is actually used to convert phenols with electron-withdrawing substituents into anilines. Due to the presence of one or two electron-donating methoxy moieties, low yields could be achieved. In industrial-scale processes, the use of ammonia, high pressure, and catalysts should make it possible to obtain higher yields [36]. However, the methoxy moieties seem to prevent the Schiff base polymerization of 4-amino-3-methoxy benzaldehyde (**1**) and 4-amino-3,5-dimethoxy benzaldehyde (**2**). Attempts to convert 4-aminobenzaldehyde in the same way, resulted in an insoluble red solid. The aldehydes were converted into the corresponding oximes 4-amino-3-methoxy benzaldehyde oxime (**3**) and 4-amino-3,5-dimethoxy benzaldehyde oxime (**4**) with high yields. Finally, amines **5** and **6** could be prepared from these oximes with moderate yields.

DSC analyses using DGEBA as the epoxy resin showed that amines **5** and **6** exhibit similar curing temperatures to the cold-curing IPDA. Due to the fact that the melting process and the curing reaction are superimposed when using amine **5**, an unsteady heat flow can be observed. TG-MS analyses demonstrated that the thermal stability of the polymer formed with the novel amine **6** was similar to those formed with IPDA, whereas the thermal stability of the polymer formed with amine **5** was significantly lower.

## Figures and Tables

**Table 1:** Onset temperatures of the curing reaction and the glass transition temperatures (T_g_) of the polymers formed by the investigated amines with DGEBA.

Amine Hardener	T_onset_ Curing	T_g_
**5**	71 °C	68 °C
**6**	79 °C	160 °C
IPDA	86 °C	112 °C
DDS	175 °C	181 °C
ABA	73 °C	137 °C

## Data Availability

The data presented in this study are available in the present article and in the related Supplementary Materials.

## Supplementary Materials

The following supporting information can be downloaded at: https://www.mdpi.com/article/10.3390/polym15040990/s1, Figure S1: Melting Point of **1**; Figure S2: ^1^H NMR spectrum of **1**; Figure S3: ^13^C {^1^H} NMR spectrum of **1**; Figure S4: Mass spectrum of **1**; Figure S5: IR spectrum of **1**; Figure S6: Melting Point of **2**; Figure S7: ^1^H NMR spectrum of **2**; Figure S8: ^13^C {^1^H} NMR spectrum of **2**; Figure S9: Mass spectrum of **2**; Figure S10: IR spectrum of **2**; Figure S11: Melting Point of **3**; Figure S12: ^1^H NMR spectrum of **3**; Figure S13: ^13^C {^1^H} NMR spectrum of **3**; Figure S14: Mass spectrum of **3**; Figure S15: IR spectrum of **3**; Figure S16: Melting Point of **4**; Figure S17: ^1^H NMR spectrum of **4**; Figure S18: ^13^C {^1^H} NMR spectrum of **4**; Figure S19: Mass spectrum of **4**; Figure S20: IR spectrum of **4**; Figure S21: Melting Point of **5**; Figure S22: ^1^H NMR spectrum of **5**; Figure S23: ^13^C {^1^H} NMR spectrum of **5**; Figure S24: Mass spectrum of **5**; Figure S25: IR spectrum of **5**; Figure S26: ^1^H NMR spectrum of **6**; Figure S27: ^13^C {^1^H} NMR spectrum of **6**; Figure S28: Mass spectrum of **6**; Figure S29: IR spectrum of **6**.
