# Supplementary material for: Synthesis, Curing and Thermal Behavior of Amine Hardeners from Potentially Renewable Sources
Source: Polymers (Basel). 2023 Feb 16;15(4):990. doi: 10.3390/polym15040990 (PMC9962791; doi:10.3390/polym15040990)
Supplement: Supplementary file 1 [file polymers-15-00990-s001.zip › polymers-2200830-supplementary.pdf]

Supporting Information

Synthesis, Curing and Thermal Behavior of Amine Hardeners from Potentially Renewable Sources

Torben Wiegand * and Andrea Osburg

F. A. Finger-Institute for Building Material Engineering, Professorship of Construction Chemistry and Polymer Materials, Bauhaus-Universität Weimar, Coudraystraße 11A, 99423 Weimar, Germany
andrea.osburg@uni-weimar.de
* Correspondence: torben.wiegand@uni-weimar.de

Contents

Characterization data of 4-amino-3-methoxy benzaldehyde (**1**) 2
- Melting Point of **1** 2
- ^{1}H NMR spectrum of **1** 3
- ^{13}C {^{1}H} NMR spectrum of **1** 4
- Mass spectrum of **1** 5
- IR spectrum of **1** 6

Characterization data 4-amino-3,5-dimethoxy benzaldehyde (**2**) 7
- Melting Point of **2** 7
- ^{1}H NMR spectrum of **2** 8
- ^{13}C {^{1}H} NMR spectrum of **2** 9
- Mass spectrum of **2** 10
- IR spectrum of **2** 11

Characterization data of 4-amino-3-methoxy benzaldehyde oxime (**3**) 12
- Melting Point of **3** 12
- ^{1}H NMR spectrum of **3** 13
- ^{13}C {^{1}H} NMR spectrum of **3** 14
- Mass spectrum of **3** 15
- IR spectrum of **3** 16

Characterization data of 4-amino-3,5-dimethoxy benzaldehyde oxime (**4**) 17
- Melting Point of **4** 17
- ^{1}H NMR spectrum of **4** 18
- ^{13}C {^{1}H} NMR spectrum of **4** 19
- Mass spectrum of **4** 20
- IR spectrum of **4** 21

Characterization data of 4-amino-3-methoxy benzylamine (**5**) 22
- Melting Point of **5** 22
- ^{1}H NMR spectrum of **5** 23
- ^{13}C {^{1}H} NMR spectrum of **5** 24
- Mass spectrum of **5** 25
- IR spectrum of **5** 26

Characterization data of 4-amino-3,5-dimethoxy benzylamine (**6**) 27
- ^{1}H NMR spectrum of **6** 27
- ^{13}C {^{1}H} NMR spectrum of **6** 28
- Mass spectrum of **6** 29
- IR spectrum of **6** 30

Characterization data of 4-amino-3-methoxy benzaldehyde (**1**)

Melting Point of **1**

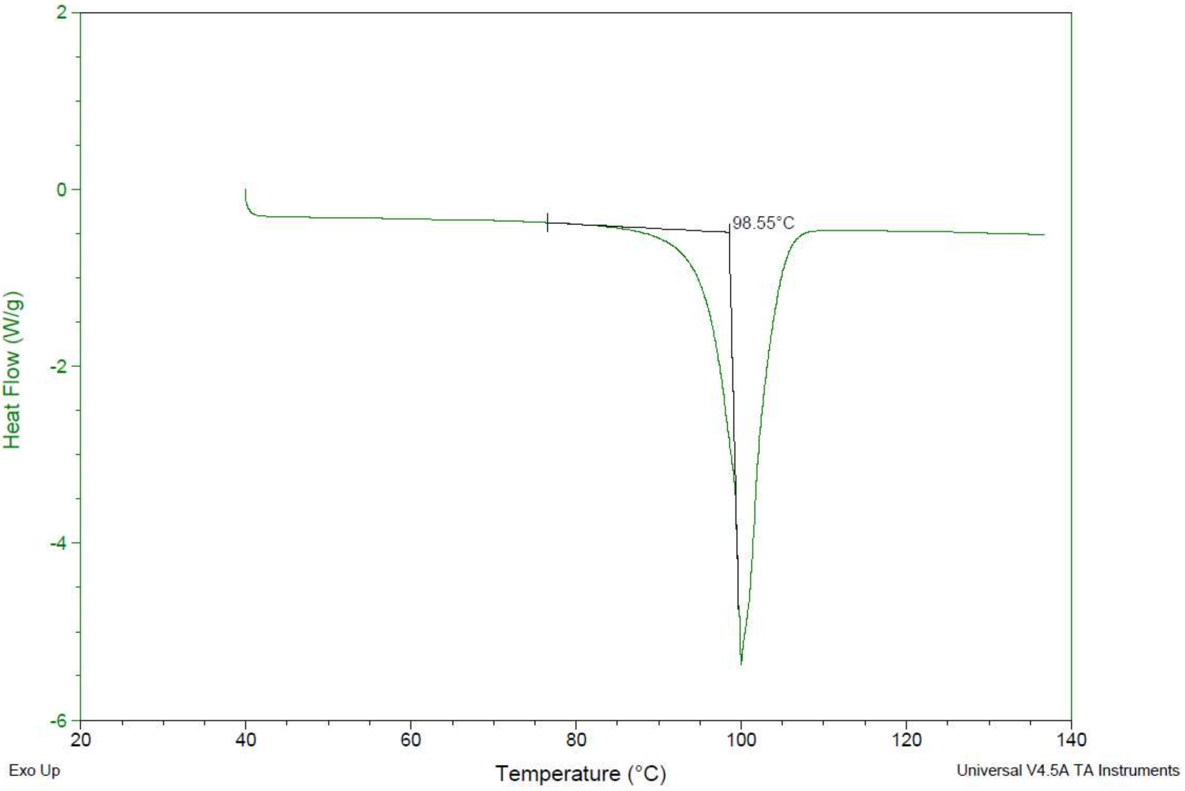

*Figure S1: Melting Point of **1**.*

^{1}H NMR spectrum of **1**

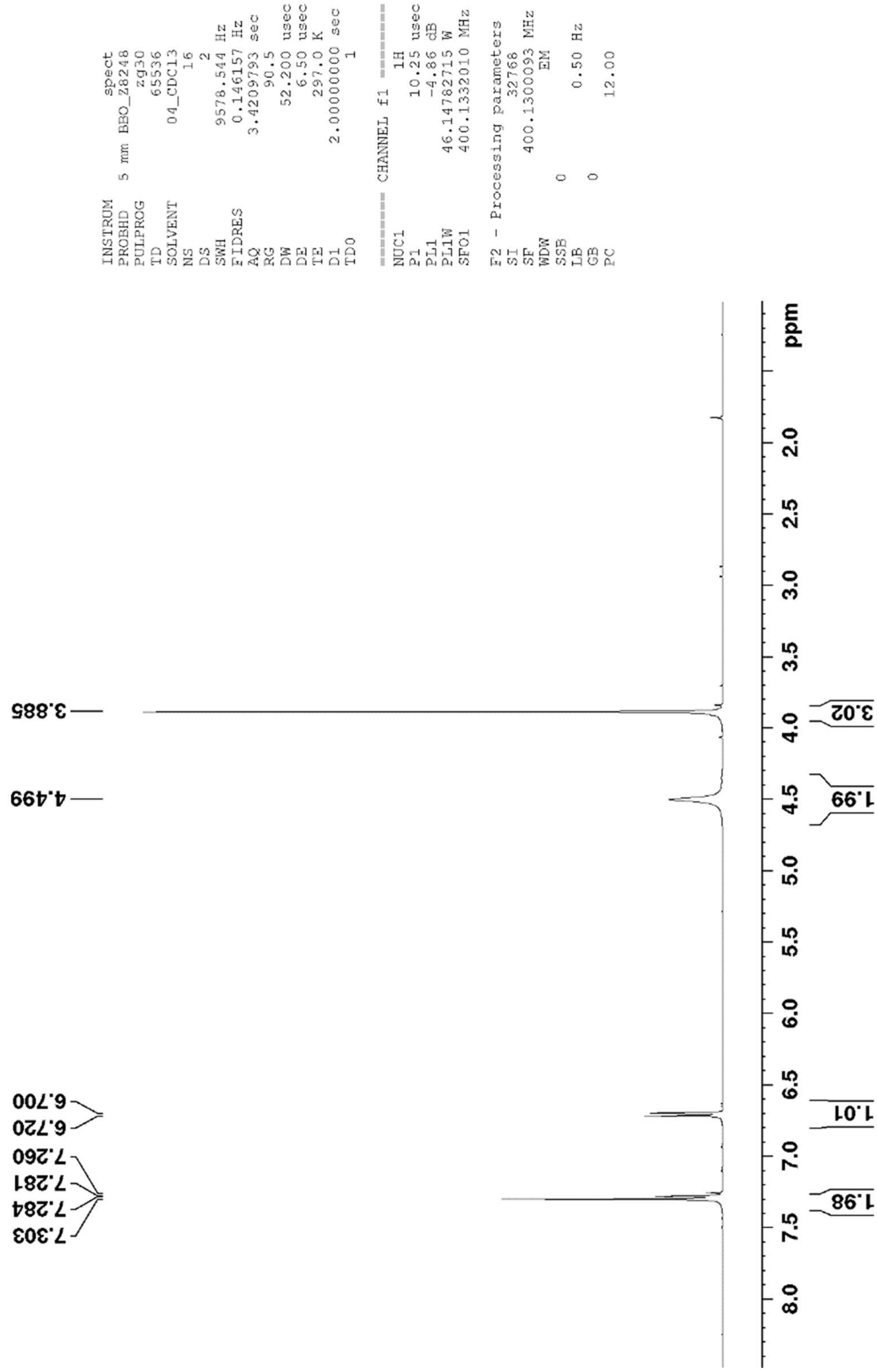

*Figure S2: ^{1}H NMR spectrum of **1**.*

^{13}C {^{1}H} NMR spectrum of **1**

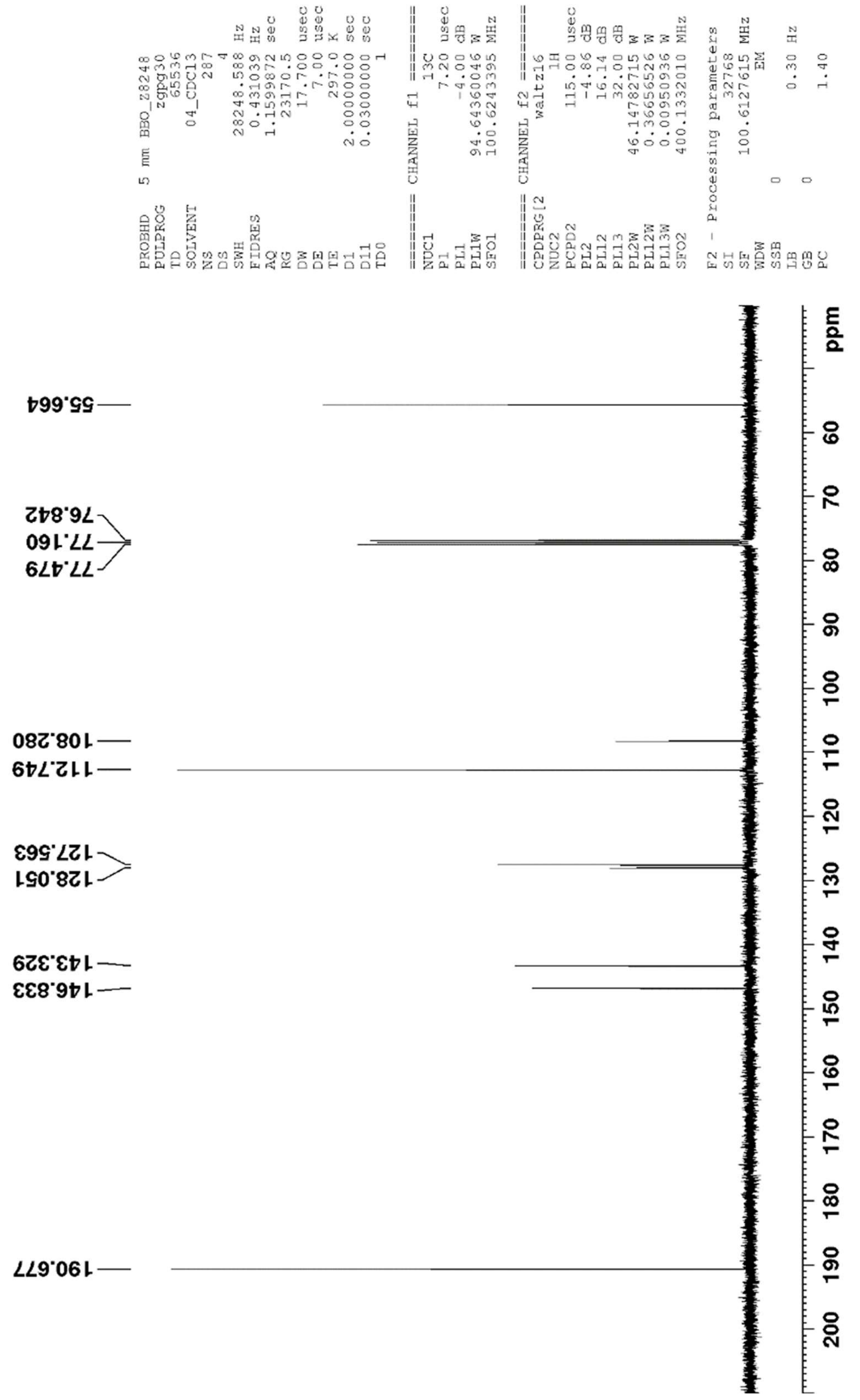

*Figure S3: ^{13}C {^{1}H} NMR spectrum of **1**.*

Mass spectrum of **1**

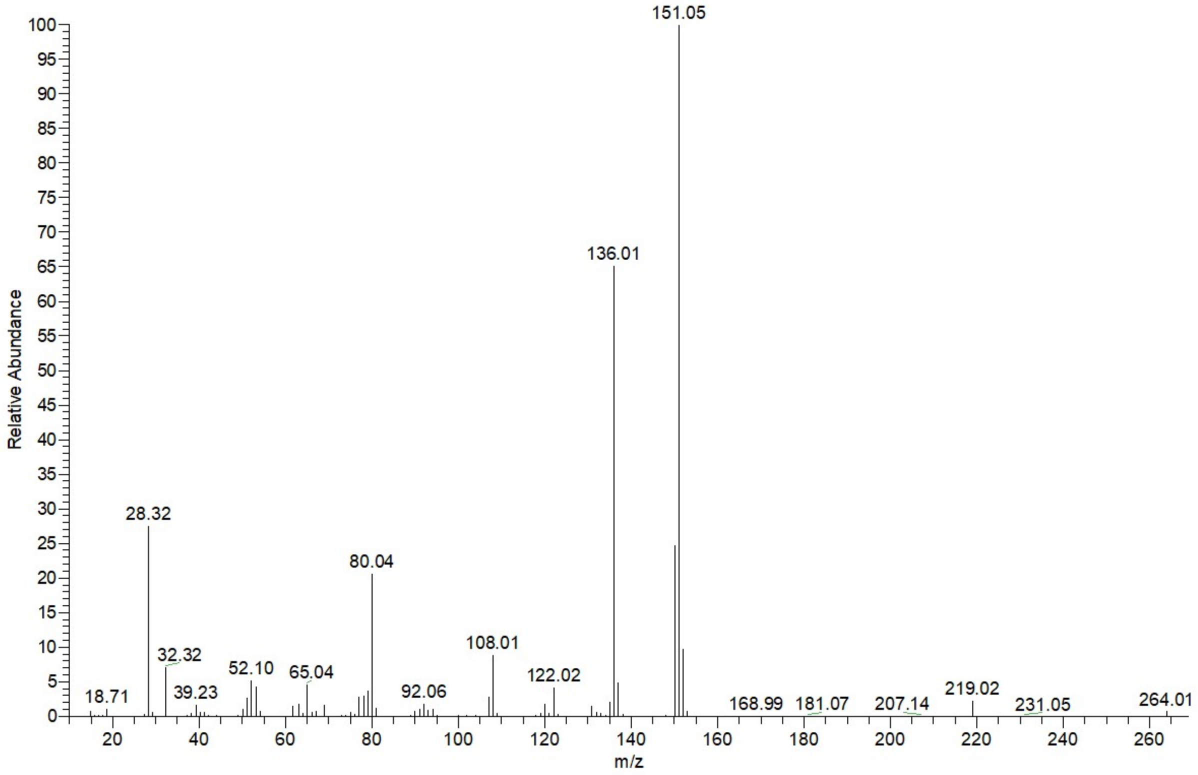

*Figure S4: Mass spectrum of **1**.*

IR spectrum of **1**

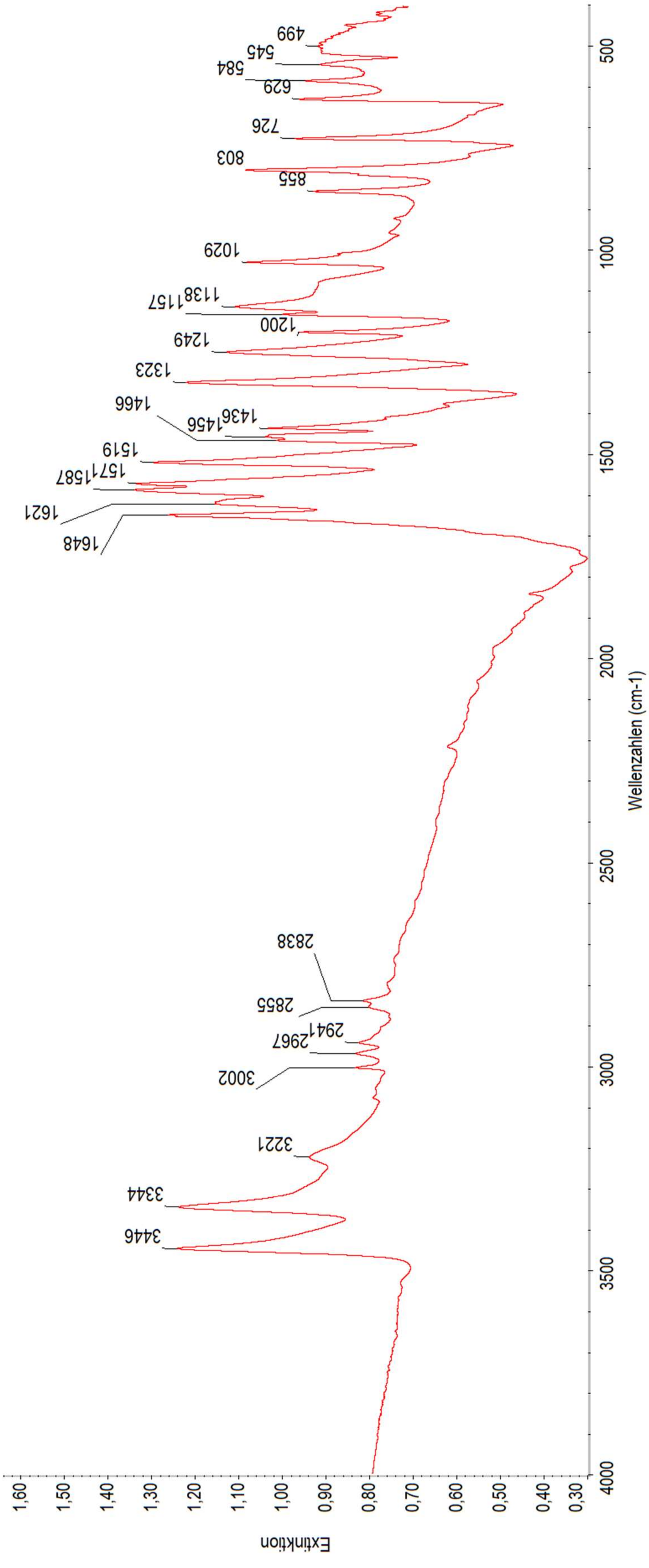

*Figure S5: IR spectrum of **1**.*

Characterization data 4-amino-3,5-dimethoxy benzaldehyde (**2**)

Melting Point of **2**

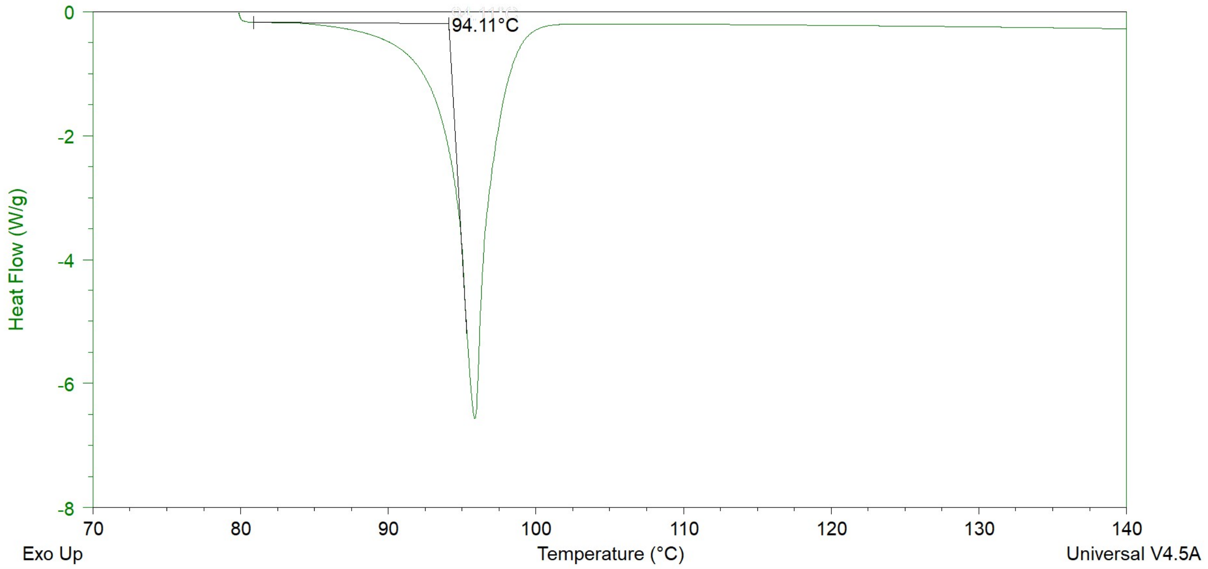

*Figure S6: Melting Point of **2**.*

^{1}H NMR spectrum of **2**

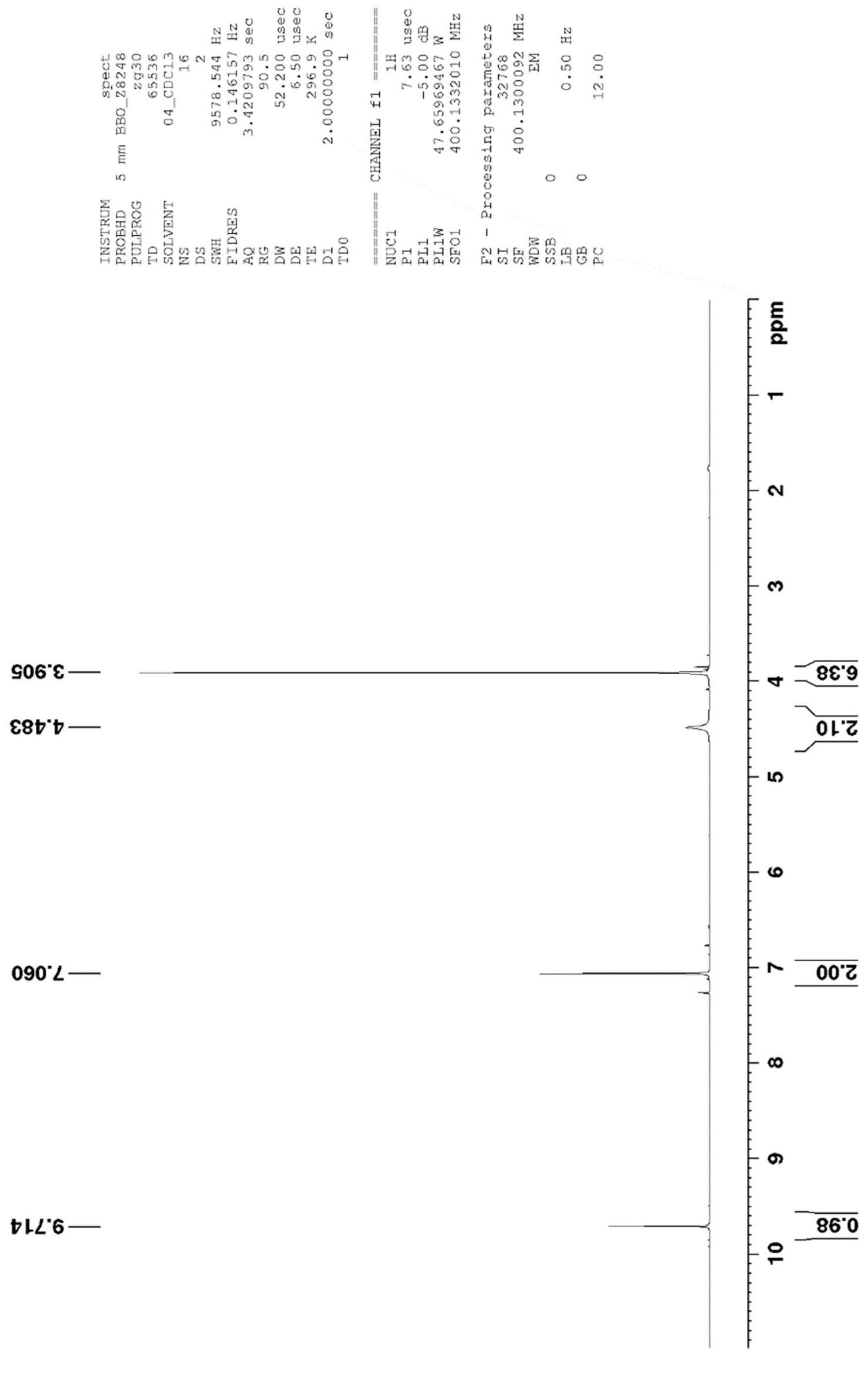

*Figure S7: ^{1}H NMR spectrum of **2**.*

^{13}C {^{1}H} NMR spectrum of **2**

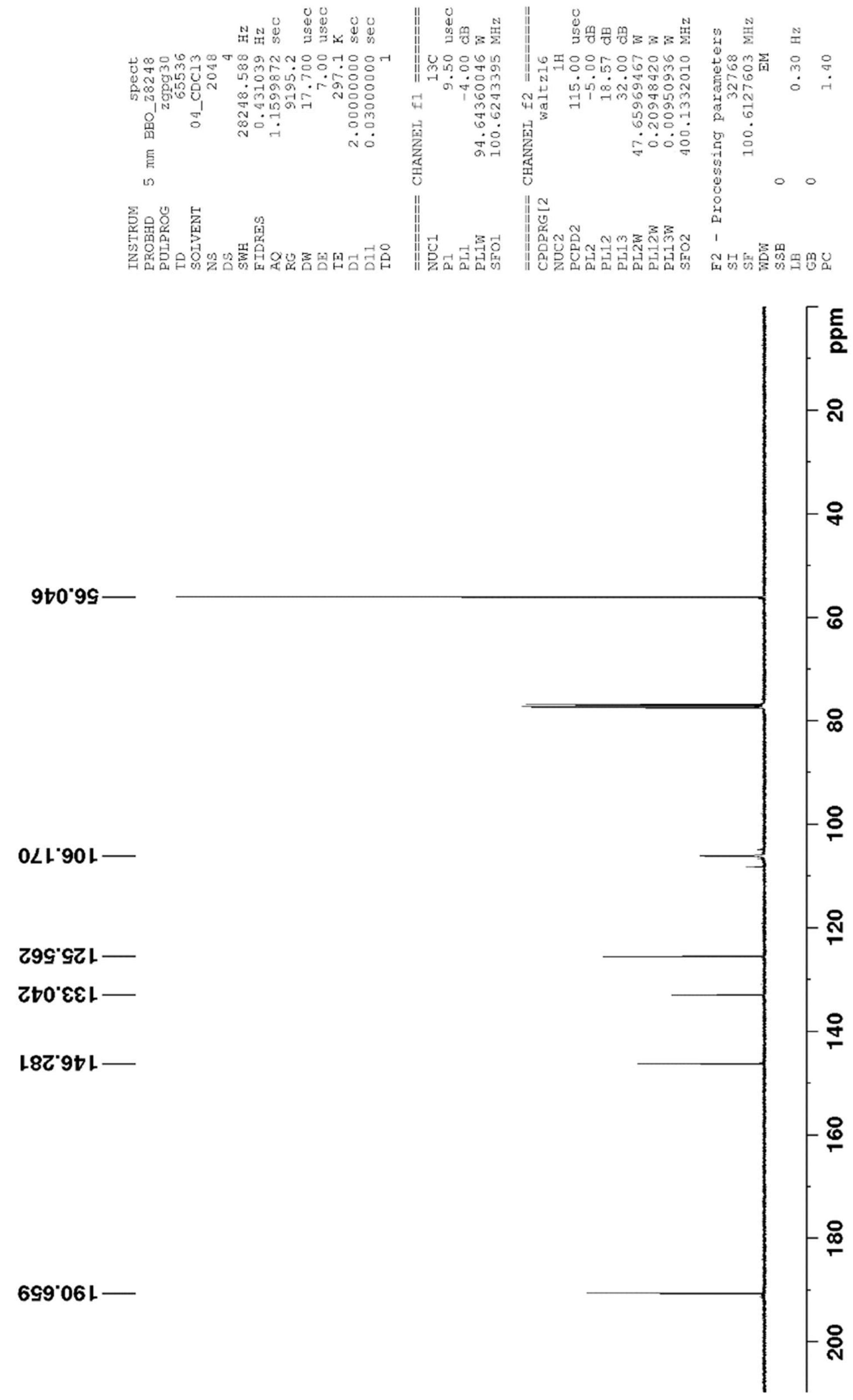

*Figure S8: ^{13}C {^{1}H} NMR spectrum of **2**.*

Mass spectrum of **2**

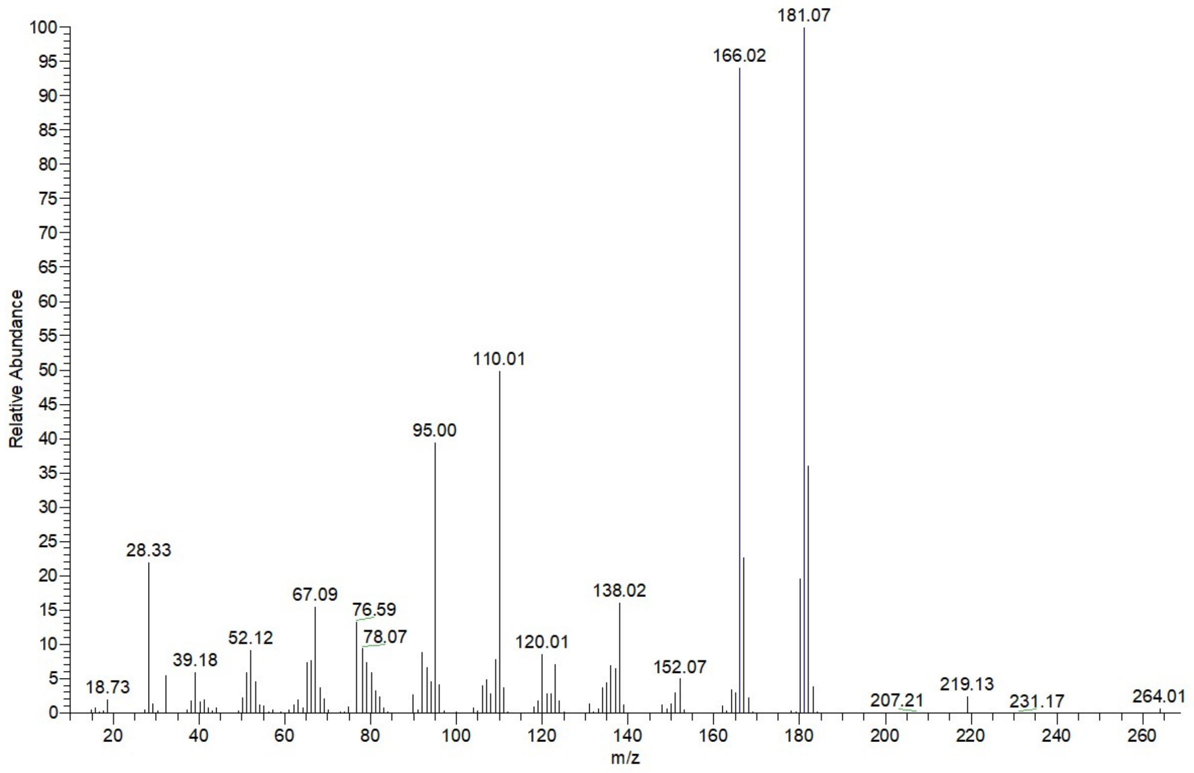

*Figure S9: Mass spectrum of **2**.*

IR spectrum of **2**

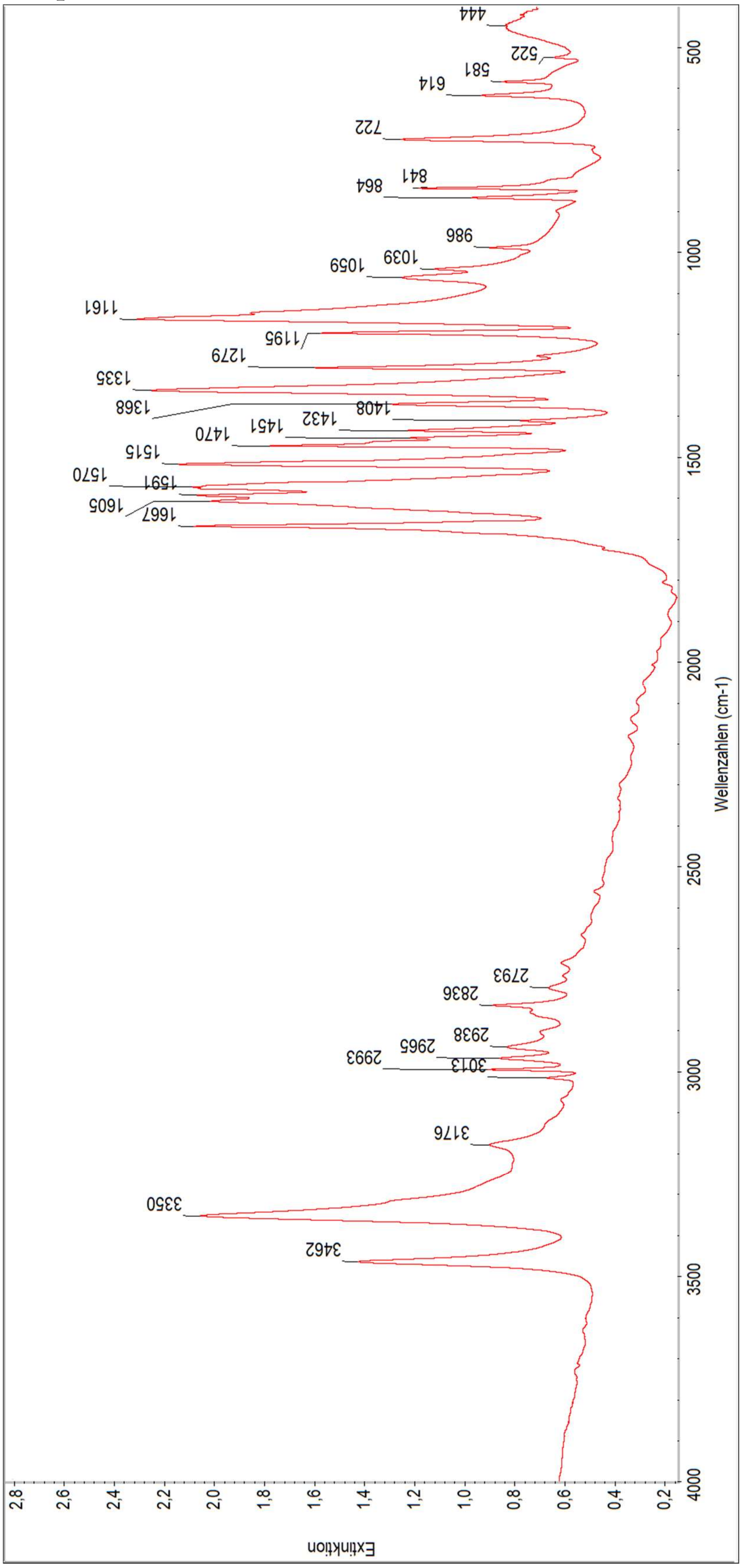

*Figure S10: IR spectrum of **2**.*

Characterization data of 4-amino-3-methoxy benzaldehyde oxime (**3**)

Melting Point of **3**

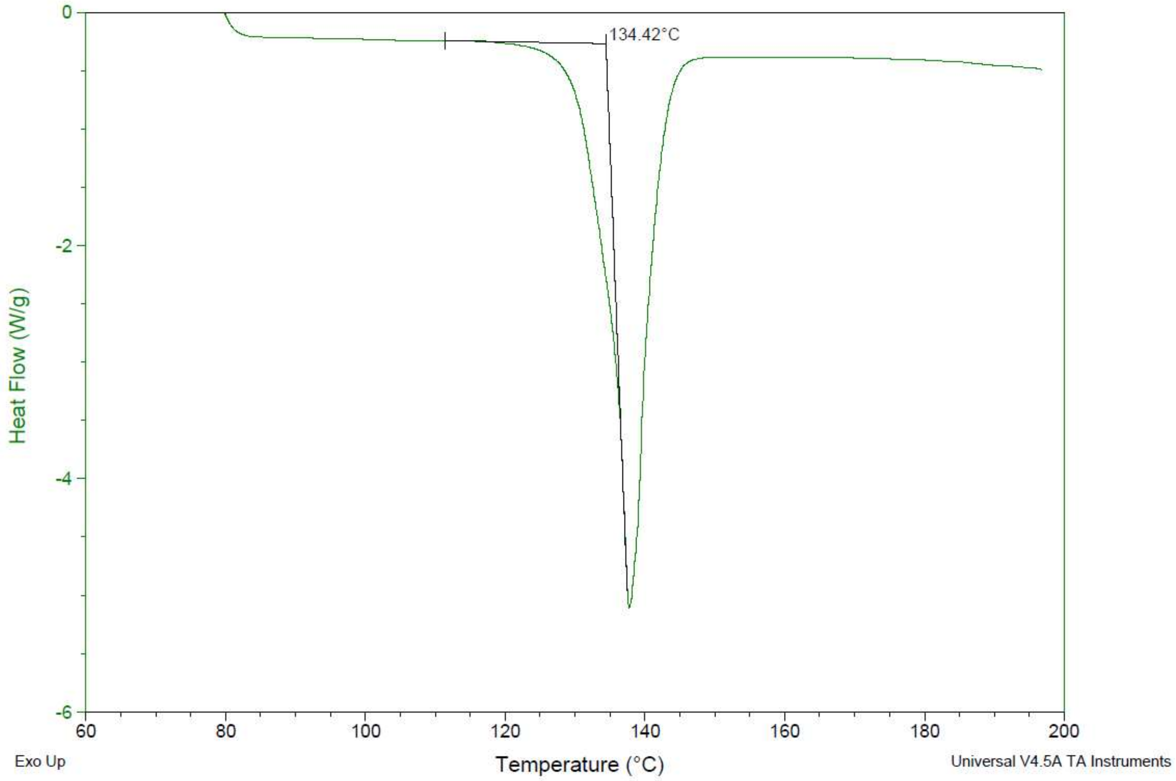

*Figure S11: Melting Point of **3**.*

^{1}H NMR spectrum of **3**

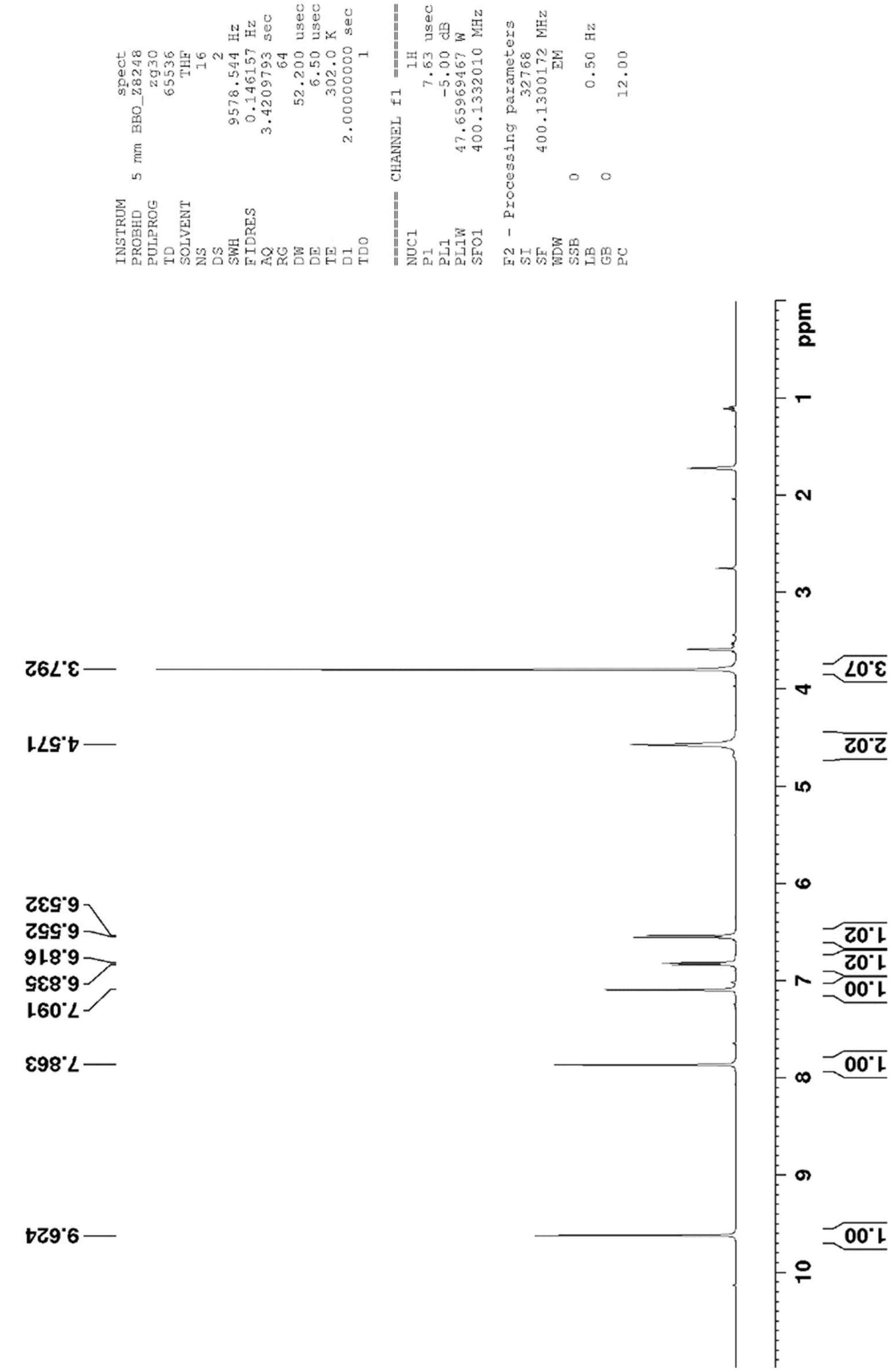

*Figure S12: ^{1}H NMR spectrum of **3**.*

^{13}C {^{1}H} NMR spectrum of **3**

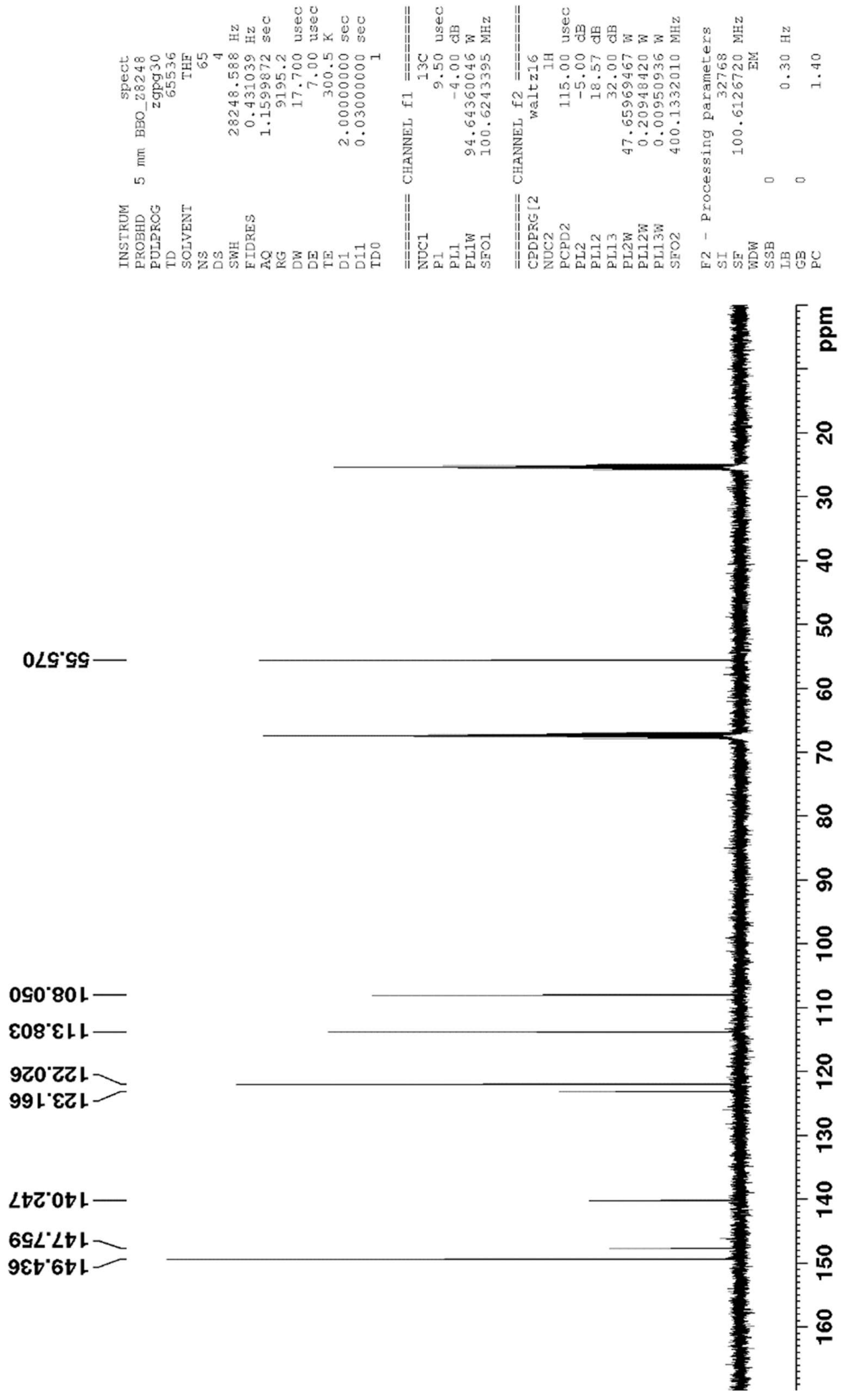

*Figure S13: ^{13}C {^{1}H} NMR spectrum of **3**.*

Mass spectrum of **3**

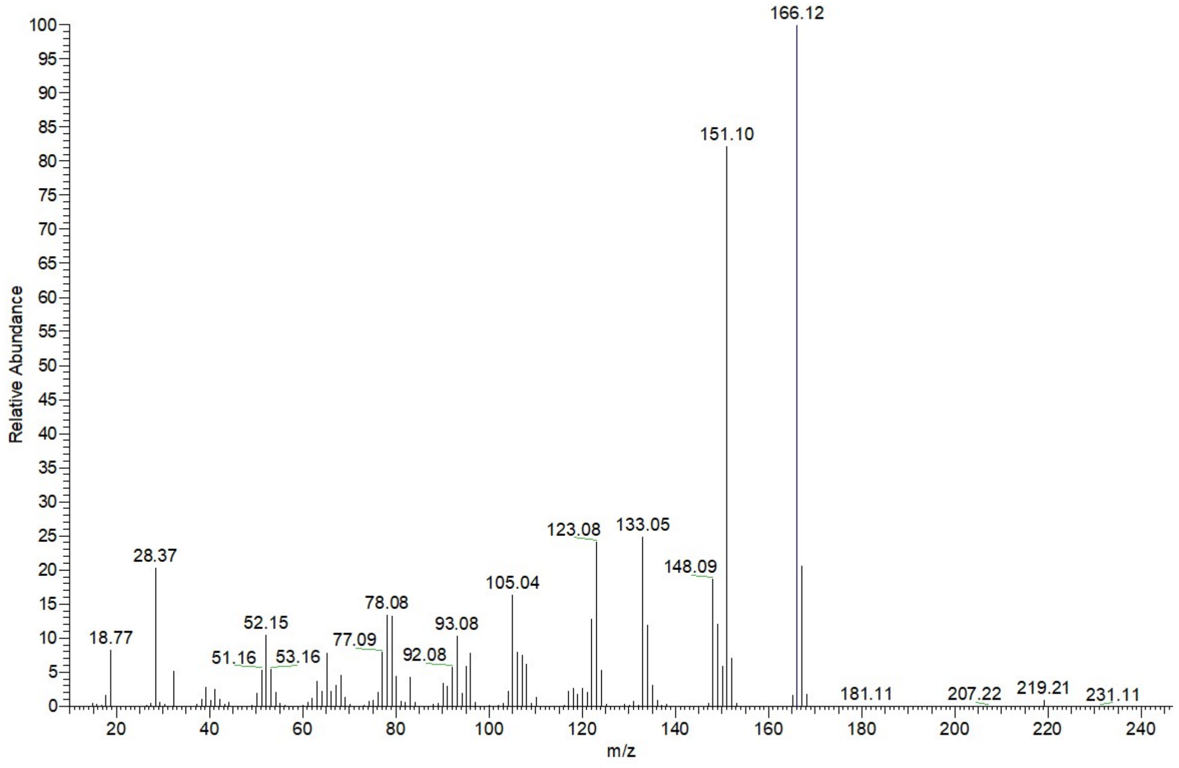

*Figure S14: Mass spectrum of **3**.*

IR spectrum of **3**

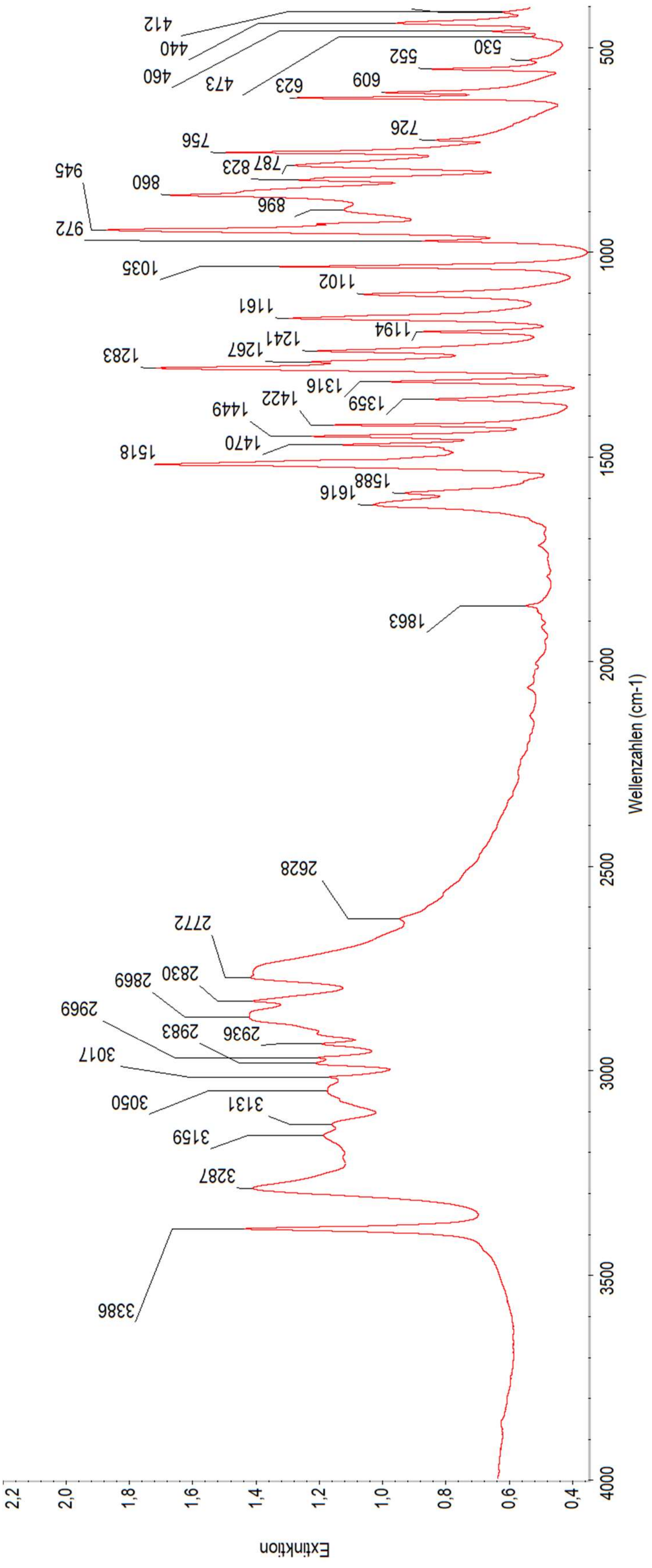

*Figure S15: IR spectrum of **3**.*

Characterization data of 4-amino-3,5-dimethoxy benzaldehyde oxime (**4**)

Melting Point of **4**

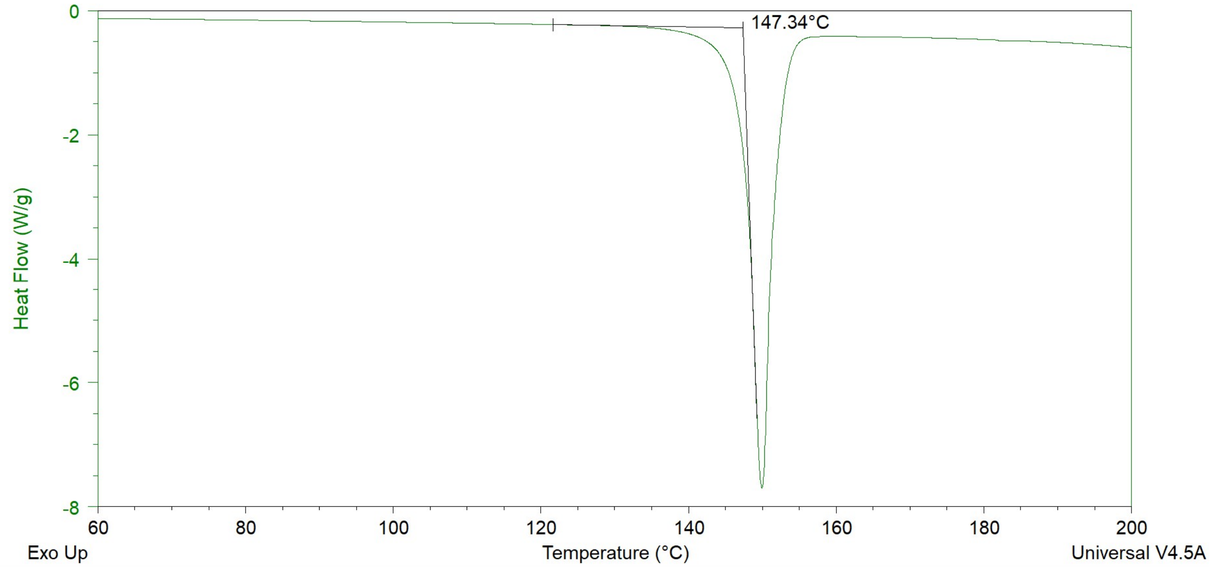

*Figure S16: Melting Point of **4**.*

^{1}H NMR spectrum of **4**

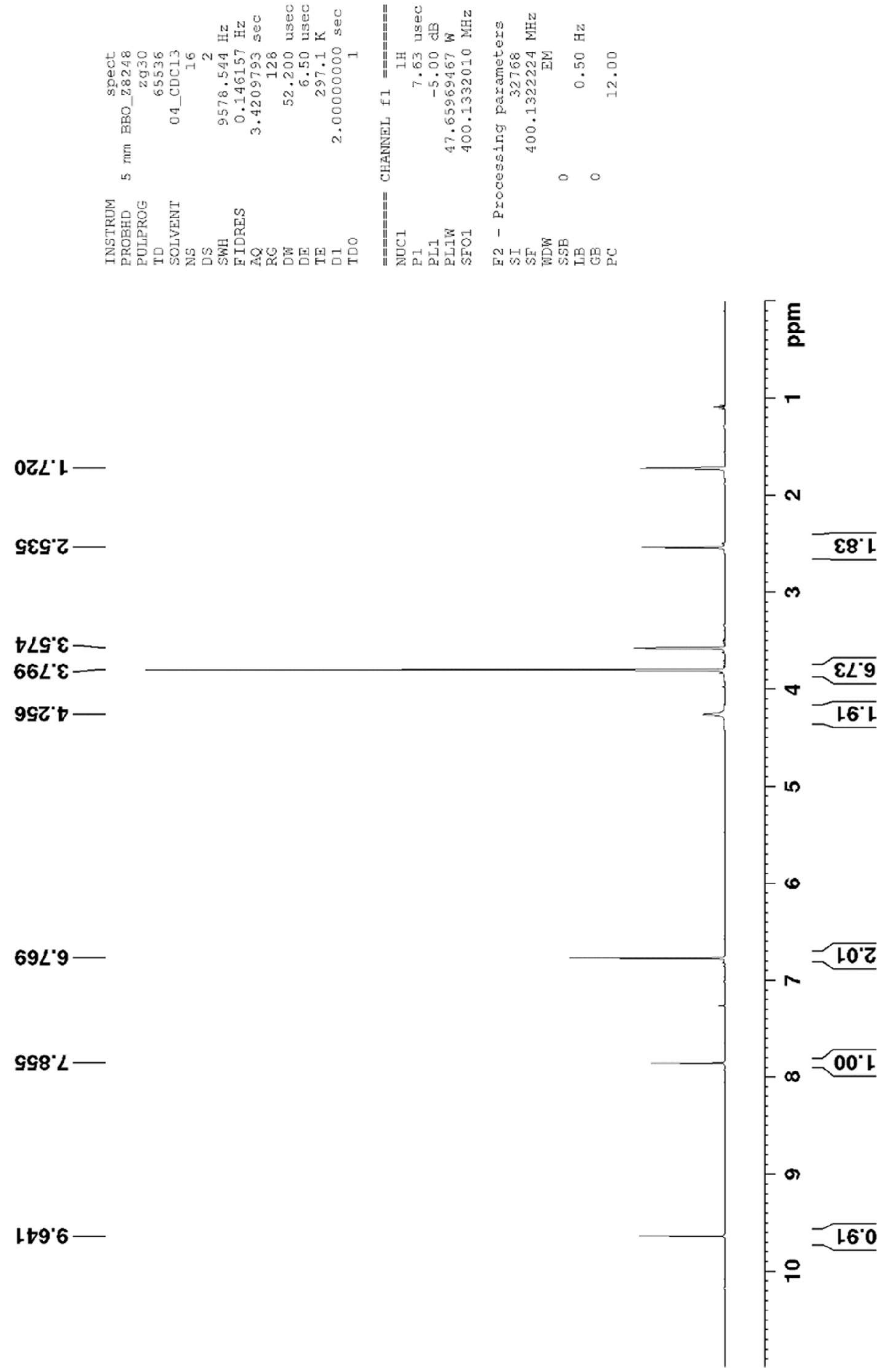

*Figure S17: ^{1}H NMR spectrum of **4**.*

^{13}C {^{1}H} NMR spectrum of **4**

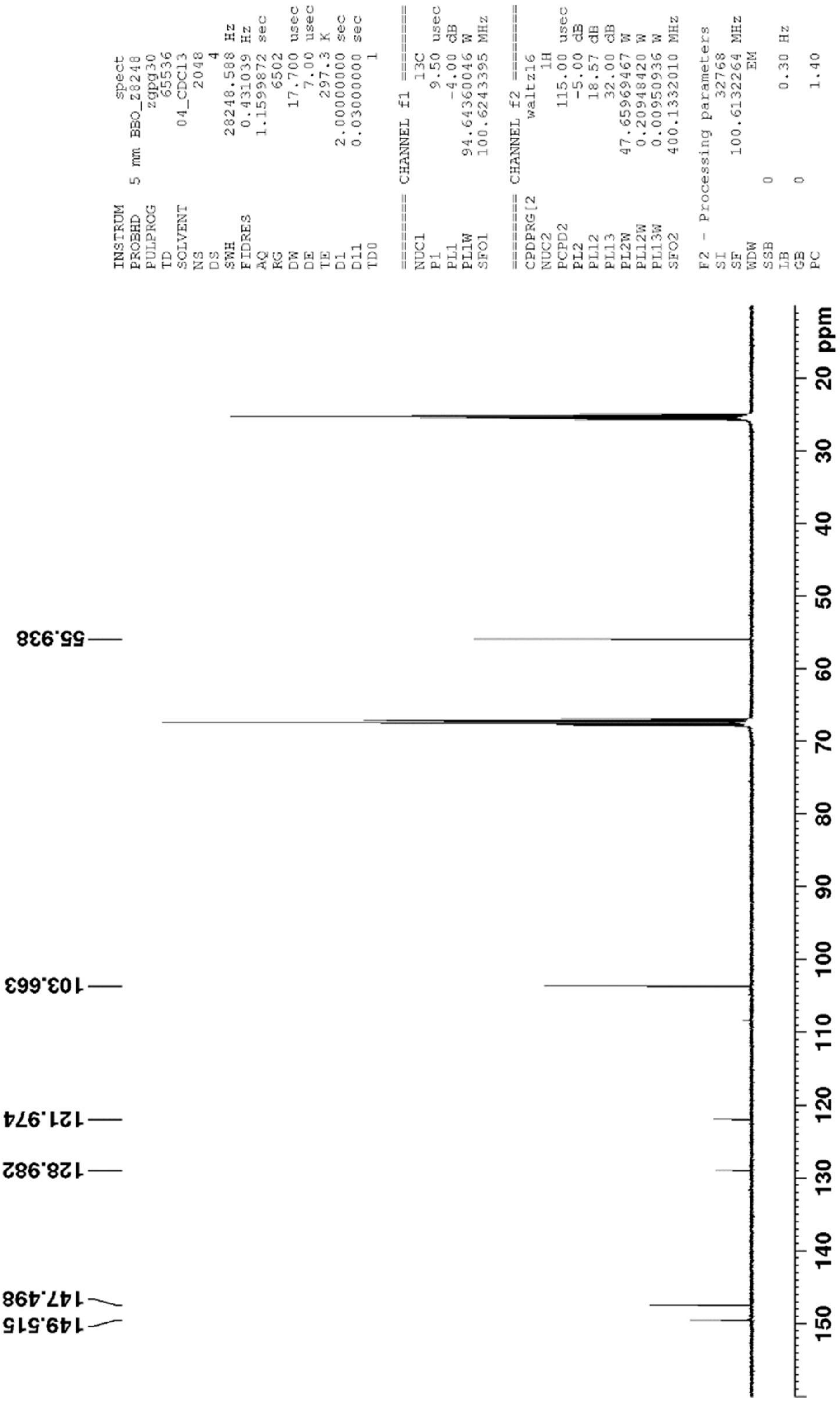

*Figure S18: ^{13}C {^{1}H} NMR spectrum of **4**.*

Mass spectrum of **4**

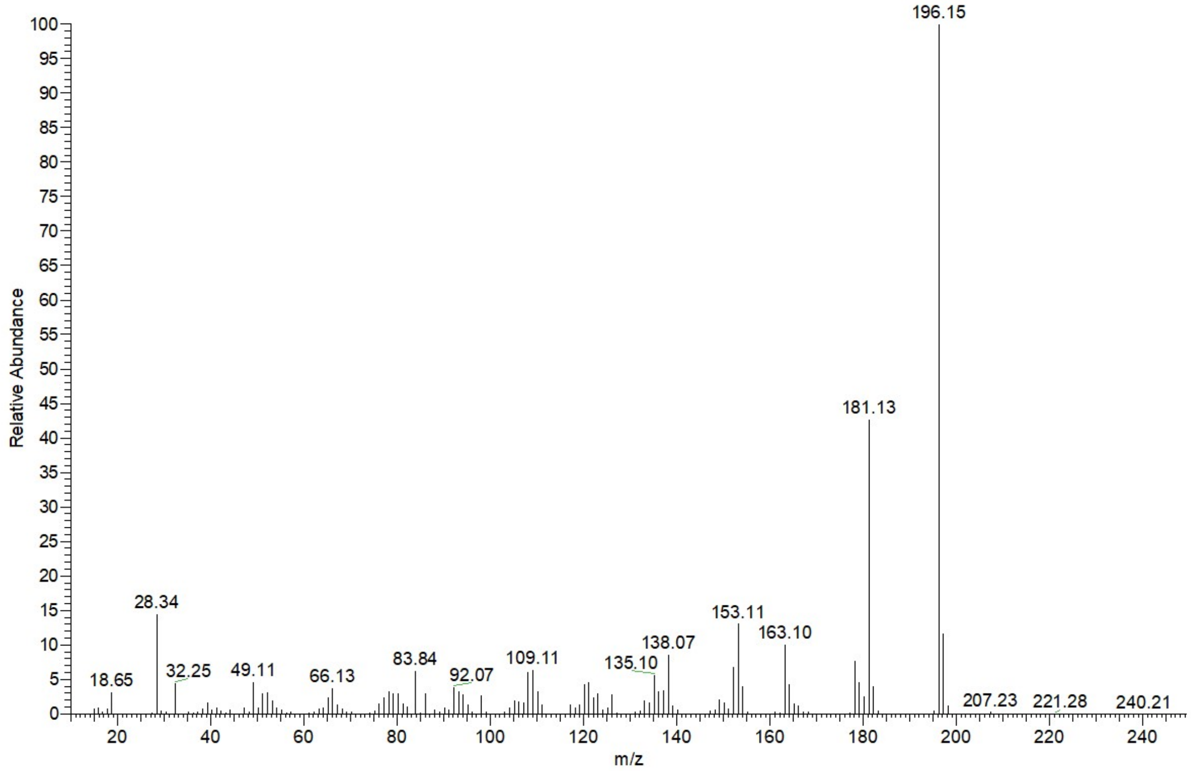

*Figure S19: Mass spectrum of **4**.*

IR spectrum of **4**

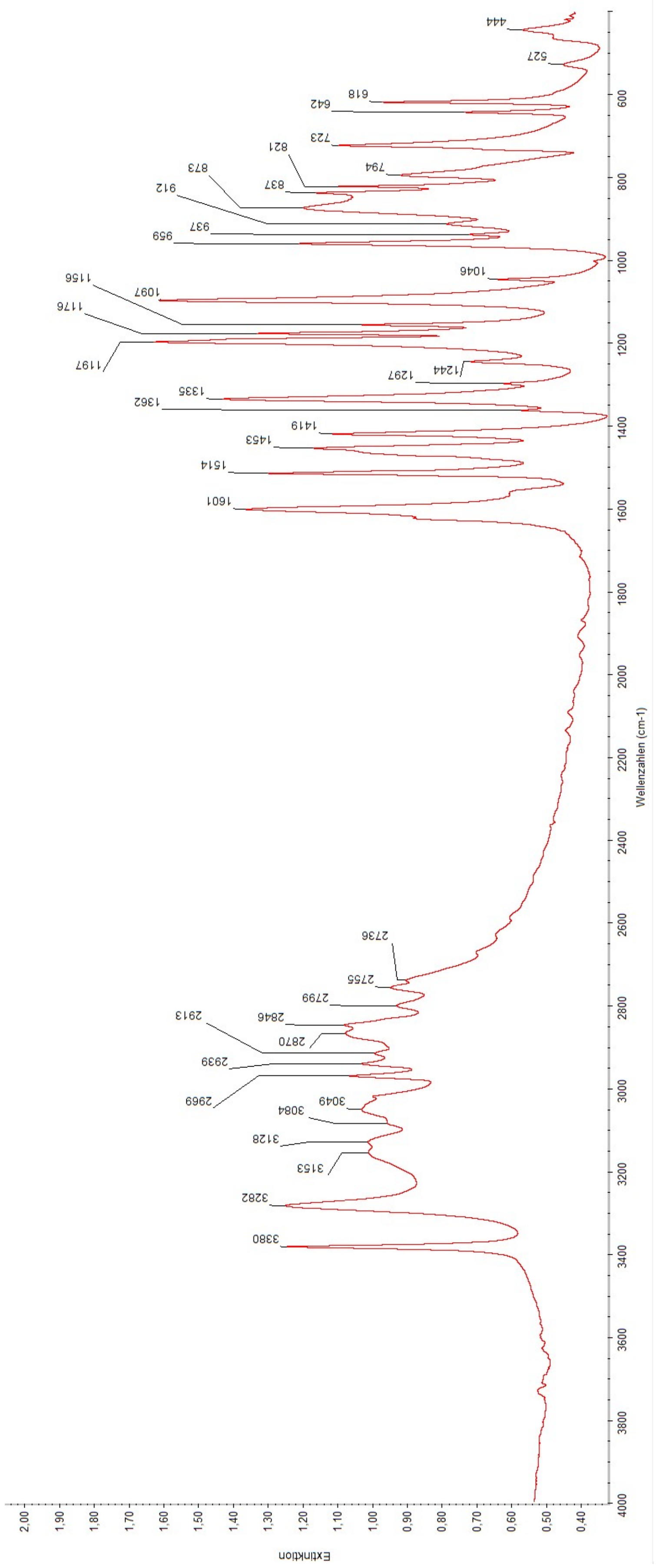

*Figure S20: IR spectrum of **4**.*

Characterization data of 4-amino-3-methoxy benzylamine (**5**)

Melting Point of **5**

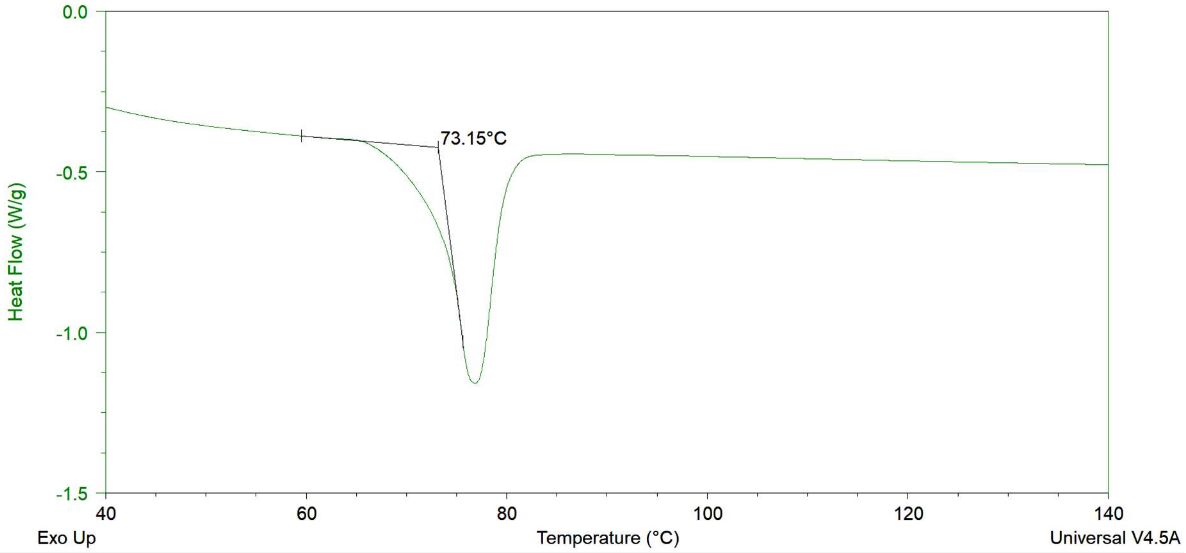

*Figure S21: Melting Point of **5**.*

^{1}H NMR spectrum of **5**

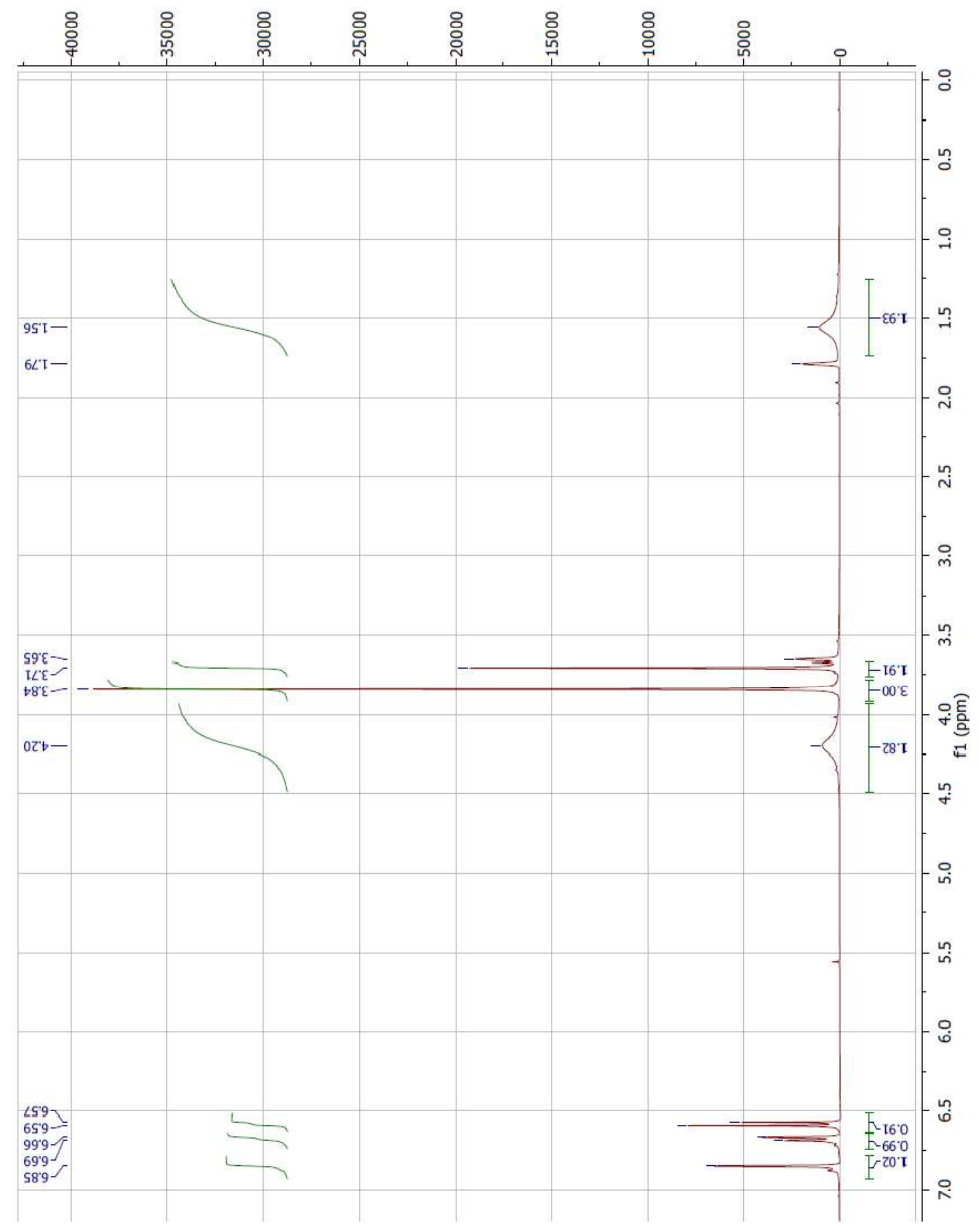

*Figure S22: ^{1}H NMR spectrum of **5**.*

^{13}C {^{1}H} NMR spectrum of **5**

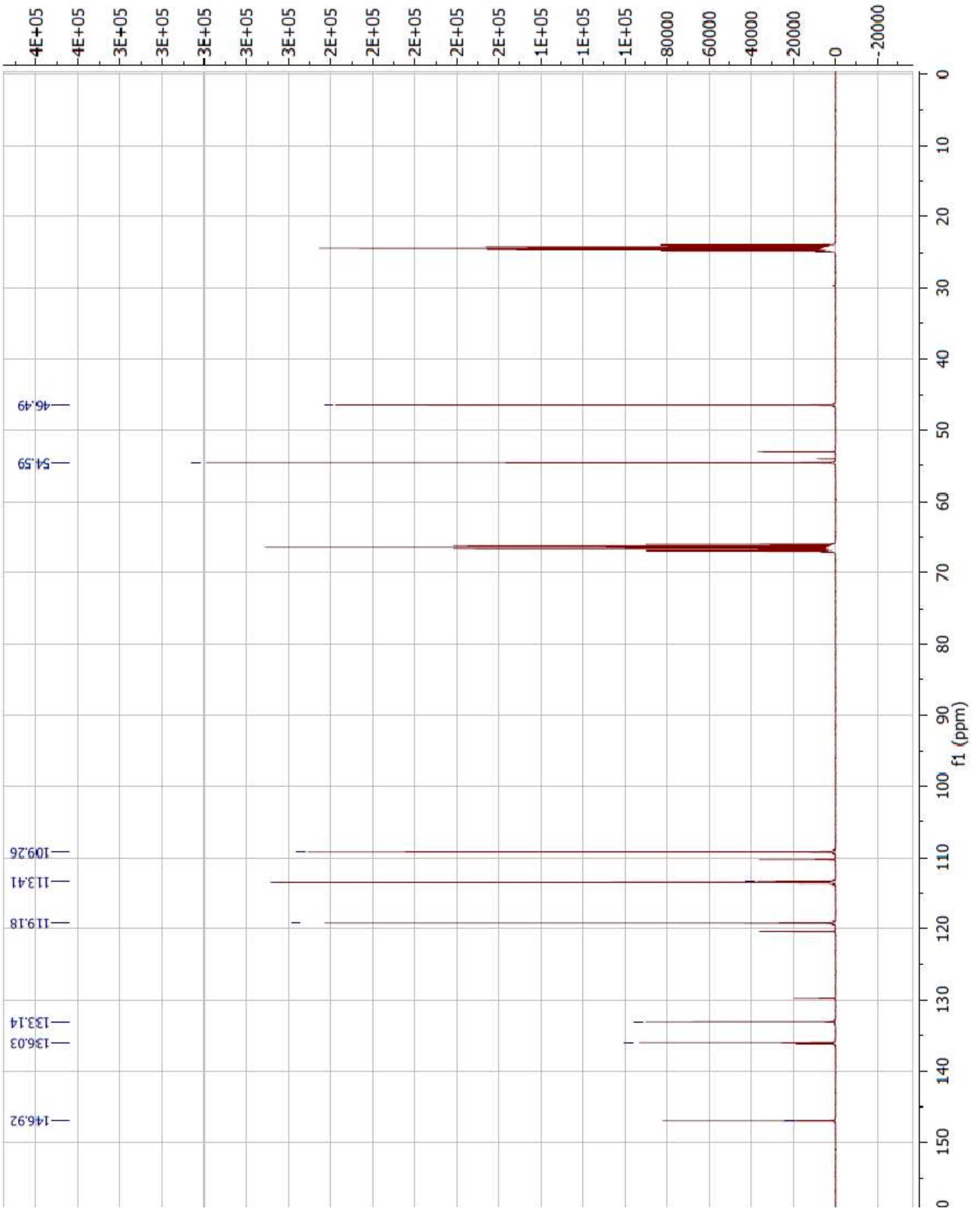

*Figure S23: ^{13}C {^{1}H} NMR spectrum of **5**.*

Mass spectrum of **5**

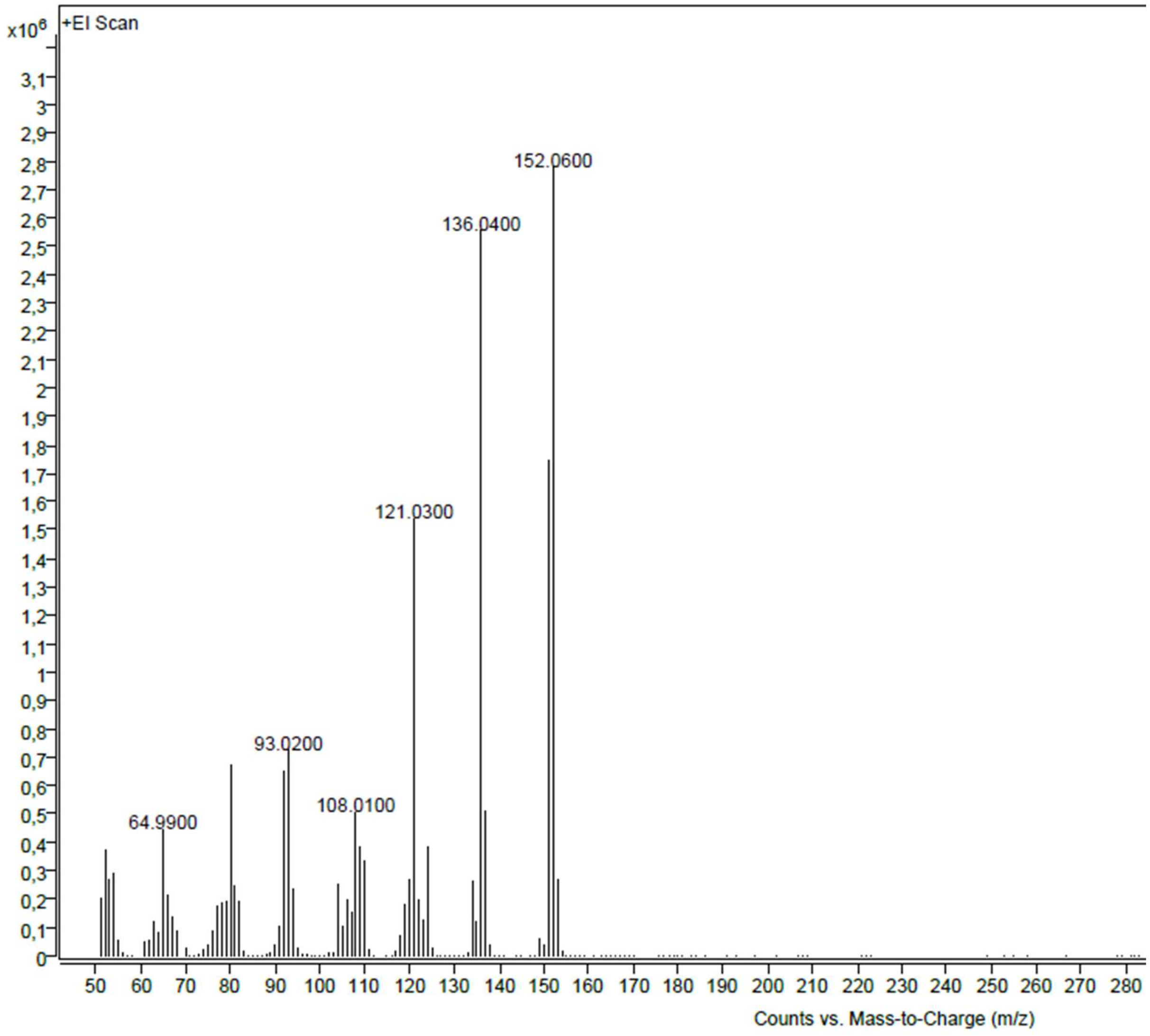

*Figure S24: Mass spectrum of **5**.*

IR spectrum of **5**

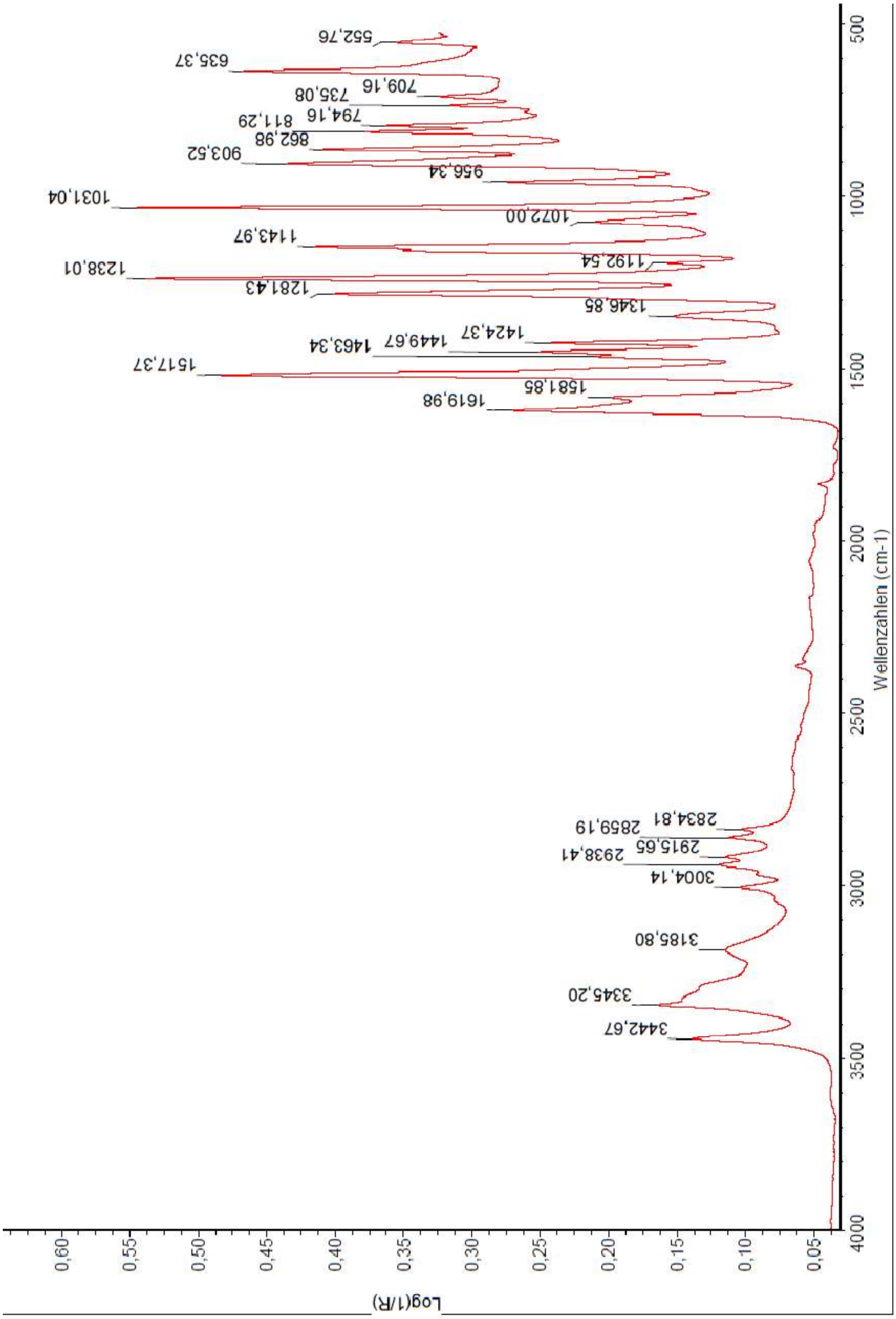

*Figure S25: IR spectrum of **5**.*

Characterization data of 4-amino-3,5-dimethoxy benzylamine (**6**)

^{1}H NMR spectrum of **6**

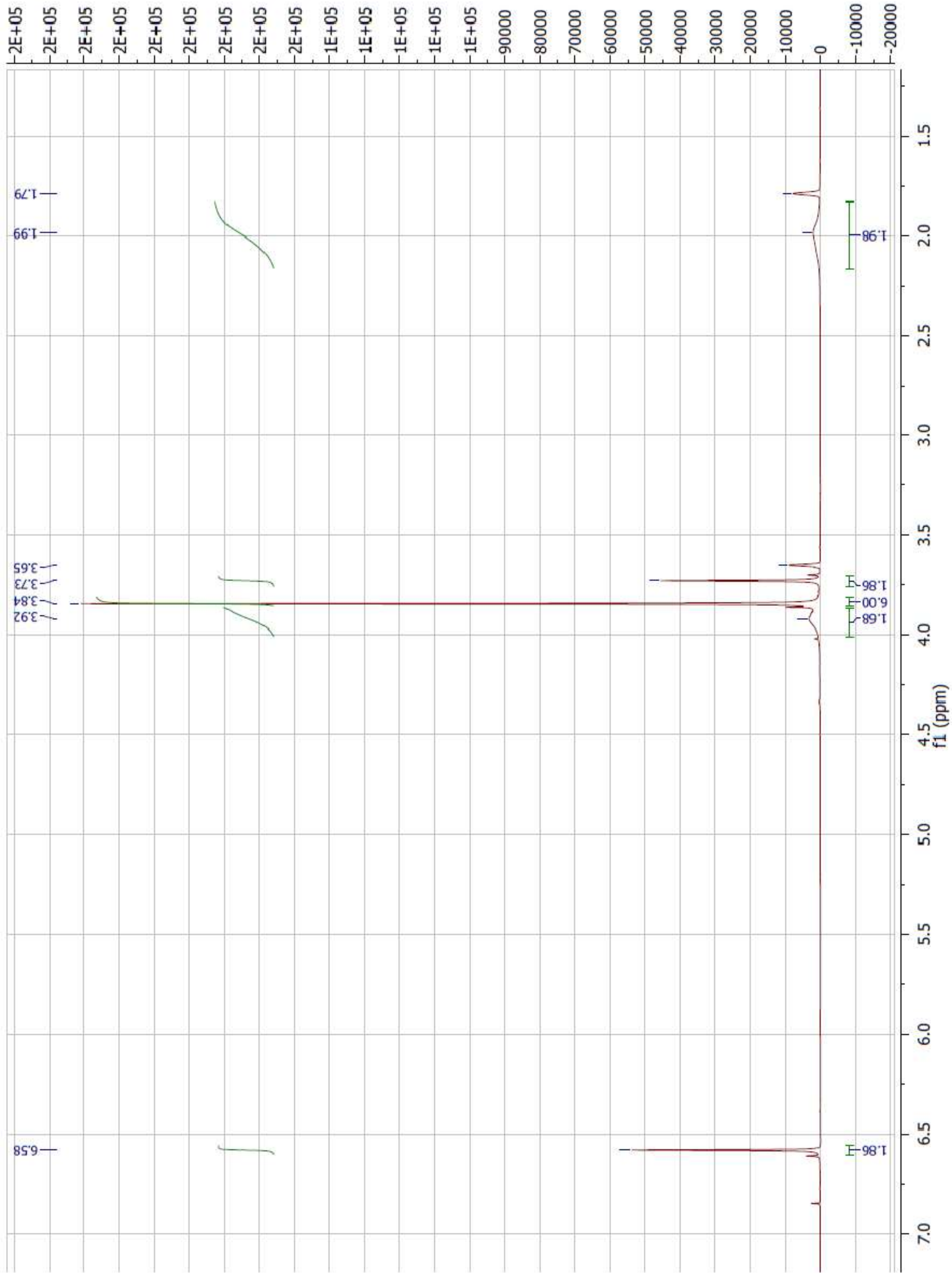

*Figure S26: ^{1}H NMR spectrum of **6**.*

^{13}C {^{1}H} NMR spectrum of **6**

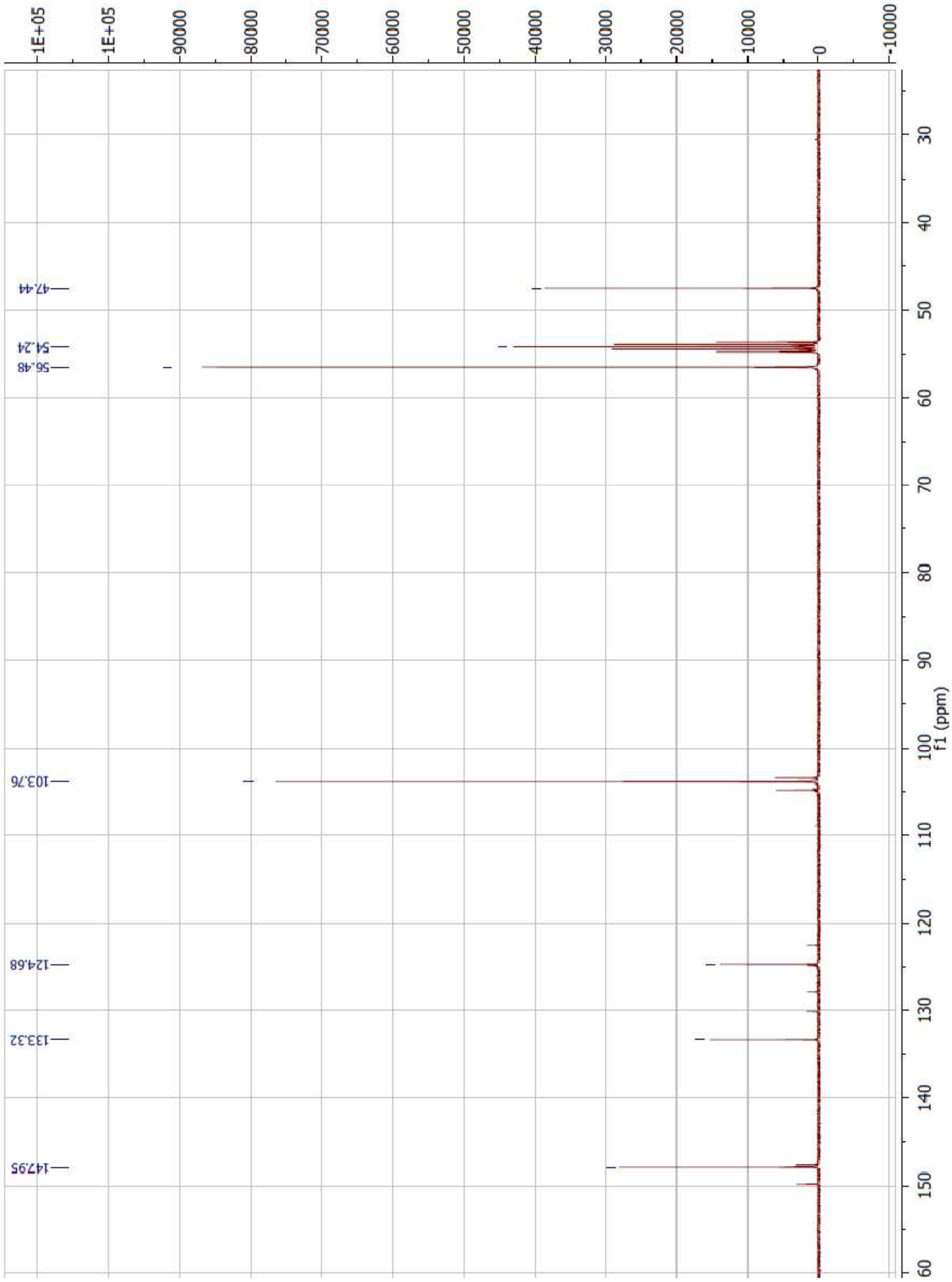

*Figure S27: ^{13}C {^{1}H} NMR spectrum of **6**.*

Mass spectrum of **6**

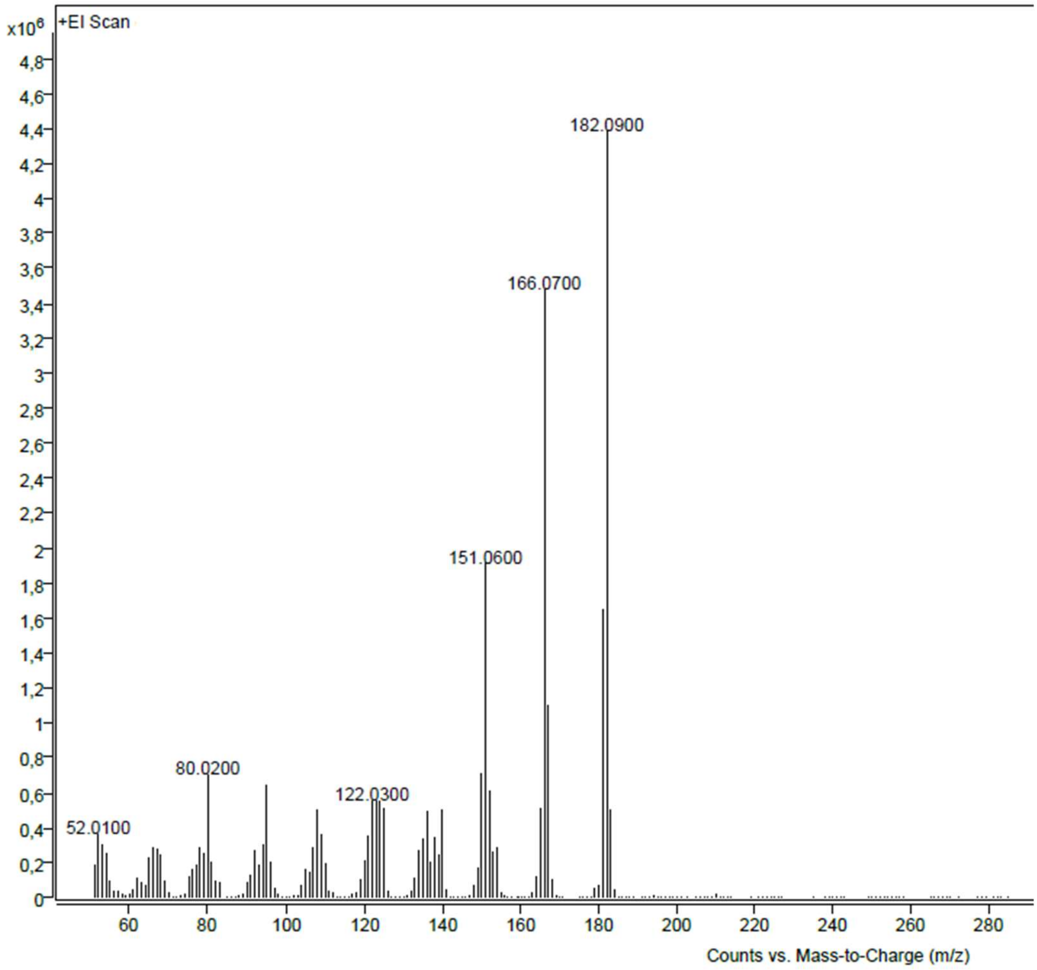

*Figure S28: Mass spectrum of **6**.*

IR spectrum of **6**

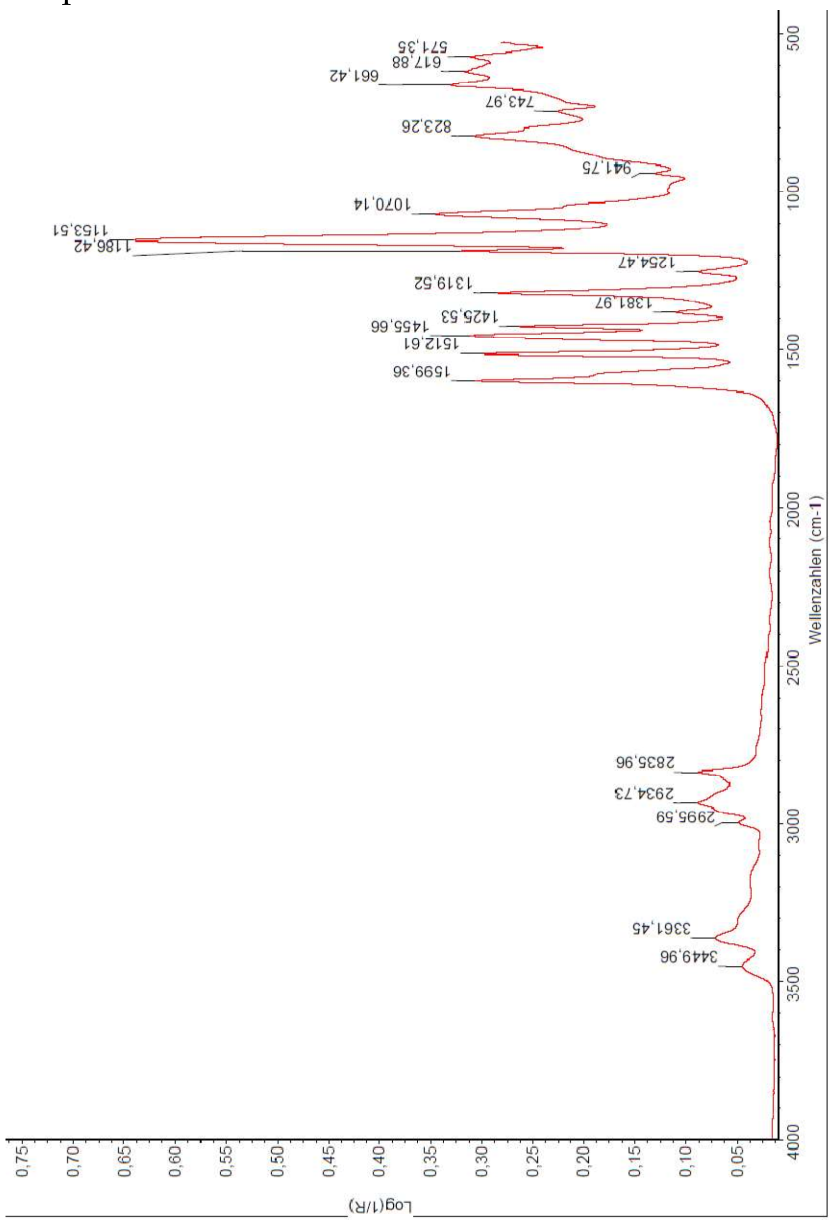

*Figure S29: IR spectrum of **6**.*
